# Developmental role of PHD2 in the pathogenesis of pseudohypoxic pheochromocytoma

**DOI:** 10.1530/ERC-21-0211

**Published:** 2021-09-20

**Authors:** Luise Eckardt, Maria Prange-Barczynska, Emma J Hodson, James W Fielding, Xiaotong Cheng, Joanna D C C Lima, Samvid Kurlekar, Gillian Douglas, Peter J Ratcliffe, Tammie Bishop

**Affiliations:** 1 Target Discovery Institute, University of Oxford, Oxford, UK; 2 Institute of Physiology and Pathophysiology, University of Heidelberg, Heidelberg, Germany; 3 Ludwig Institute for Cancer Research, University of Oxford, Oxford, UK; 4 The Francis Crick Institute, London, UK; 5 The Department of Experimental Medicine and Immunotherapeutics, University of Cambridge, Cambridge, UK; 6 BHF Centre of Research Excellence, Division of Cardiovascular Medicine, Radcliffe Department of Medicine, John Radcliffe Hospital, University of Oxford, Oxford, UK

**Keywords:** PHD, HIF, hypoxia, adrenal medulla, pheochromocytoma

## Abstract

Despite a general role for the HIF hydroxylase system in cellular oxygen sensing and tumour hypoxia, cancer-associated mutations of genes in this pathway, including *PHD2*, *PHD1*, *EPAS1* (encoding HIF-2α) are highly tissue-restricted, being observed in pseudohypoxic pheochromocytoma and paraganglioma (PPGL) but rarely, if ever, in other tumours. In an effort to understand that paradox and gain insights into the pathogenesis of pseudohypoxic PPGL, we constructed mice in which the principal HIF prolyl hydroxylase, *Phd2*, is inactivated in the adrenal medulla using TH-restricted Cre recombinase. Investigation of these animals revealed a gene expression pattern closely mimicking that of pseudohypoxic PPGL. Spatially resolved analyses demonstrated a binary distribution of two contrasting patterns of gene expression among adrenal medullary cells. *Phd2* inactivation resulted in a marked shift in this distribution towards a *Pnmt*^−^/*Hif-2α*^+^/*Rgs5*^+^ population. This was associated with morphological abnormalities of adrenal development, including ectopic TH^+^ cells within the adrenal cortex and external to the adrenal gland. These changes were ablated by combined inactivation of *Phd2* with *Hif-2α*, but not *Hif-1α*. However, they could not be reproduced by inactivation of *Phd2* in adult life, suggesting that they arise from dysregulation of this pathway during adrenal development. Together with the clinical observation that pseudohypoxic PPGL manifests remarkably high heritability, our findings suggest that this type of tumour likely arises from dysregulation of a tissue-restricted action of the PHD2/HIF-2α pathway affecting adrenal development in early life and provides a model for the study of the relevant processes.

## Introduction

Pheochromocytoma and paraganglioma (PPGL) are tumours of the autonomic paraganglia that arise in diverse anatomical locations from the skull base to the pelvis. Those found within the adrenal glands (AGs) are known as pheochromocytoma (PCC) and those in extra-adrenal structures including the carotid body are commonly termed paraganglioma (PGL). Molecular analysis of these tumours has revealed a number of subtypes or clusters, with distinct patterns of gene expression within the tumour being associated with different groups of tumour-associated mutations. Genetic profiling has revealed four such subtypes: kinase signalling, Wnt-altered, cortical admixture and pseudohypoxia (Crona* et al.* 2017, Fishbein & Wilkerson 2018). The pseudohypoxia subtype (or Cluster I) is associated with mutations affecting transcriptional pathways induced by hypoxia.

The transcriptional response to hypoxia is mediated by hypoxia-inducible factor (HIF) (reviewed in Bishop & Ratcliffe 2014), a heterodimer consisting of an oxygen-regulated α and a constitutively expressed β subunit. HIF-α is regulated by a series of 2-oxoglutarate-dependent dioxygenases that generate an oxygen-dependent signal by hydroxylation of specific prolyl residues in HIF-α subunits which are targeted for proteasomal degradation via the E3 ubiquitin ligase von Hippel-Lindau (VHL) protein. Mammalian species have multiple HIF-α isoforms, of which HIF-1α and HIF-2α are the most abundant and best studied. They also express three closely related isoforms of the HIF prolyl hydroxylase enzymes (PHD1, PHD2 and PHD3, otherwise known as EGLN2, EGLN1 and EGLN3), of which PHD2 is the most abundant and important regulator of HIF.

HIF is commonly activated in cancer and its role in oncogenesis has attracted widespread interest, particularly in view of the recent development of drugs with the potential to activate or inactivate components of the pathway therapeutically (reviewed in Semenza 2019, Choueiri & Kaelin 2020). Nevertheless, this relationship of HIF activation to oncogenesis has proved more complex than anticipated. For instance, although HIF is commonly upregulated in cancer, direct genetic activation by mutation of any of the key components of the pathway is rare in most forms of cancer.

The pseudohypoxic subtype of PPGLs is an important exception. These tumours manifest a ‘pseudohypoxic’ pattern of gene expression encompassing upregulation of certain HIF target genes, together with alterations in the expression of differentiation markers (Dahia* et al.* 2005, Favier* et al.* 2009, 2012, Waldmann* et al.* 2010, Burnichon* et al.* 2011, Lorenzo* et al.* 2013, Toledo* et al.* 2013, Welander* et al.* 2014, Yang* et al.* 2015, Fishbein* et al.* 2017). They are associated with loss-of-function mutations in *VHL*, *PHD2* and *PHD1* and gain-of-function mutations in *HIF-2α* (otherwise known as *EPAS1*) as well as mutations in genes encoding the tricarboxylic acid cycle enzymes succinate dehydrogenase (*SDHB*/*D*/*C*/*A* or *SDHx*) and fumarate hydratase (*FH*) (Dahia* et al.* 2005, Ladroue* et al.* 2008, Zhuang* et al.* 2012, Yang* et al.* 2015). Impaired function of the latter two enzymes leads to the accumulation of succinate and fumarate, respectively, which are able to inhibit 2-oxoglutarate-dependent dioxygenases including the HIF prolyl hydroxylases. All these mutations therefore have the potential to activate HIF, suggesting that in this setting it is HIF that provides the oncogenic drive.

However, there are a number of puzzling features in these associations. First, PPGLs in general manifest an unusually high ratio of inherited to sporadic forms. For instance, up to 40% of PPGLs are associated with germline or post-zygotic but very early somatic mutation, as assessed by family history or distribution of mutant cells (Buffet* et al.* 2020). Secondly, the spectrum of gene dysregulation in pseudohypoxic PPGLs does not align exactly with that of dynamically regulated HIF transcriptional targets (Dahia* et al.* 2005, Favier* et al.* 2009, 2012, Waldmann* et al.* 2010, Burnichon* et al.* 2011, Lorenzo* et al.* 2013, Toledo* et al.* 2013, Welander* et al.* 2014, Yang* et al.* 2015, Fishbein* et al.* 2017). Thirdly, human *VHL* mutations associated with PCCs have a complex relationship to dysregulation of HIF: type 1 *VHL* mutations, which show complete dysregulation of HIF, are not associated with pheochromocytoma whereas type 2A, B and C *VHL* mutations, which are associated with pheochromocytoma, show either less severe or no dysregulation of HIF, at least when assayed *in vitro* or in heterologous cell types (Kaelin 2008). Fourthly, *Vhl* inactivation in the adrenal medulla (AM) and carotid body of the mouse results in tissue atrophy rather than tumour formation (Macias* et al.* 2014).

In an attempt to shed light on these paradoxical findings and better understand the role of activation of HIF pathways in the AM, we have examined the effects of inactivation of the principal HIF prolyl hydroxylase *Phd2* in the AM, using Cre recombinase restricted by the tyrosine hydroxylase (TH) promoter, in the mouse. We report that TH-restricted constitutive inactivation of *Phd2* in the AM results in a ‘pseudohypoxic pattern’ of gene expression in which dynamic activation of HIF transcription is superimposed on a developmental shift in populations of AM cells manifesting specific patterns of gene expression associated with the presence or absence of phenylethanolamine *N*-methyltransferase (PNMT). Changes in gene expression were accompanied by morphological abnormalities including ectopic TH^+^ cells within the adrenal cortex and in peri-adrenal structures. These findings, together with marked differences between constitutive and inducible inactivation of *Phd2* in adult life, suggest that the pathological activation of the PHD2/HIF-2 pathway during adrenal development is critical for its tumourigenic action.

## Methods

### Ethical approval and animals

Animal experimental protocols were approved by the University of Oxford Medical Science Division Ethical Review Committee and are compliant with the UK Home Office Animals (Scientific Procedures) Act 1986. Experiments were performed on ~3 month-old mice and sex-matched controls, unless stated otherwise. Mice were kept in individually ventilated cages with free access to water and food. *Phd2^f/f^, Hif-1α^f/f^* and *Hif-2α^f/f^* alleles are as described (Cramer* et al.* 2003, Gruber* et al.* 2007, Mazzone* et al.* 2009). Note that *Phd2* is equivalent to *Egln1* and *Hif-2α* is equivalent to *Epas1*; we have used the *Phd2*/*Hif-2α* terminology to simplify mechanistic interpretation for the reader. TH^+^ cell-specific inactivation was achieved using the constitutively expressed *TH-IRES-Cre* (*THCre*; Lindeberg *et al.* 2004) or inducible *TH-IRES-CreER* (*THCreER*; Rotolo *et al.* 2008) and ubiquitous inactivation using the inducible *Rosa26Cre^ERT2^* (*RosaCreER*; Vooijs *et al.* 2001). Each mouse line was backcrossed with C57BL/6 for at least five generations.

### Drug administration

For adult-onset *Phd2* inactivation, ~6 week-old mice were dosed once daily with 2 mg tamoxifen orally (20 mg/mL in corn oil containing 10% ethanol, Sigma) for 5 consecutive days. *Phd2^f/f^;RosaCreER* and *Phd2^f/f^;THCreER* mice were sacrificed 17 days or ~3 months, respectively, after the start of treatment.

### Tissue collection

Animals were killed by an overdose of isoflurane (Piramal Critical Care, West Drayton, UK) and exsanguination from the inferior vena cava. Blood was collected using heparinised needles. AGs for RT-qPCR were dissected into ice-cold phosphate buffered saline (PBS, in mM: 137 NaCl, 2.7 KCl, 4 Na_2_H_2_PO_4_.7H_2_O, 1.5 KH_2_PO_4_) in diethyl pyrocarbonate-treated water (0.1%, v/v, Sigma). For histology, mice were perfused-fixed with 5 mL PBS followed by 5 mL 4% paraformaldehyde (PFA)/PBS (w/v) (Sigma). Dissected AGs were fixed in 4% PFA/PBS overnight, transferred into 70% (v/v) ethanol then dehydrated in an ascending ethanol series ending in histoclear (National Diagnostics, Atlanta, US), embedded in 60°C paraffin and sectioned to 4 μm thickness with a Microm HM 355S microtome (Thermo Fisher Scientific).

### Catecholamine measurements

Blood was centrifuged at 800 ***g*** for 5 min and plasma was separated and stored at −80°C. Adrenaline and noradrenaline were detected in diluted plasma samples using the Epinephrine/Norepinephrine ELISA Kit (KA1877, Abnova, Taoyuan City, Taiwan). Signal absorbance was read at 450 nm using a FLUOstar Omega microplate reader (BMG Labtech, Aylesbury, UK). Catecholamine concentrations were calculated using a linear standard curve.

### Immunohistochemistry

Sections were immunostained for TH using an EnVision+ kit (Dako Denmark A/S) with a polyclonal rabbit anti-TH antibody (1:5000, NB300-109, Novus Biologicals, Cambridge, UK) (Bishop* et al.* 2013).

### *In situ* hybridisation

mRNA was detected in sections using the manual RNAScope 2.5 HD BROWN assay or, for dual *in situ* hybridisation, the RNAScope 2.5 HD Duplex assay (Advanced Cell Diagnostics, Newark, US). RNAScope probes: Mm-Pnmt (426421 or 426421-C2 for dual RNAScope), Mm-Epas1 (314371), Mm-Rgs5 (430181), Mm-Vegfa-OI (43961). Imaging was performed with a Leica DM 1000 LED microscope (Leica Biosystems).

### Proximity ligation *in situ* hybridisation (PLISH)

Multiplex fluorescence *in situ* hybridisation was performed using PLISH (Nagendran* et al.* 2018). Sections were de-paraffinised, boiled in a 10 mM citrate buffer (pH 6.0) with 0.05% lithium dodecyl sulfate (Sigma) and processed in sealed hybridisation chambers (Grace Biolabs, Bend, US). Tissues were treated with 0.1 mg/mL Pepsin (Roche-10108057001, Sigma-Aldrich) in 0.1 M HCl, followed by (at 37°C): hybridisation of barcoded gene probes and bridge sequences, DNA ligation (10 CEU/mL T4 DNA ligase, M0202T, New England Biolabs, Ipswich, US) and extension by rolling circle amplification (1 U/mL Nxgen phi29 polymerase, Lucigen, Middleton, US). Samples were incubated with fluorophore-conjugated oligonucleotides specific to the barcode for the targeted gene probe (Supplementary Methods, see section on supplementary materials given at the end of this article), washed then treated with TruVIEW autofluoresence quenching kit (Vector Laboratories, Burlingame, US), stained with DAPI and mounted. Imaging was performed with a Zeiss Axio Imager M1 microscope (Jena, Germany) and analysed with Qupath software (Bankhead* et al.* 2017).

### Morphometric analysis

AM volume was modelled by measuring the TH^+^ area of one in eight consecutive slides of the AG using ImageJ software (NIH) (Bishop* et al.* 2008, Fielding* et al.* 2018). *In situ* hybridisation signal was quantified using trainable Weka segmentation plugin in Fiji ImageJ 1.53c software (NIH) (Cheng* et al.* 2020).

### RT-qPCR

AGs were collected from five mice per genotype and AMs sub-dissected under an SMZ-745 stereo microscope (Nikon) and stored in RNAProtect (Qiagen) on ice. Pooled tissues were homogenised in RLT+ buffer (Qiagen) using a ProScientific PRO200 Homogenizer (Cole-Parmer, Eaton Socon, UK). RNA was isolated using the RNeasy Plus Micro Kit (Qiagen), cDNA prepared using the QuantiTect RT Kit (Qiagen) and RT-qPCR performed with the TaqMan Fast Advanced Master Mix Kit (Thermo Fisher Scientific) in the StepOnePlus Real-Time PCR System (Applied Biosystems). Three technical replicates were used in each biological repeat with *Actb* serving as a reference gene (see Supplementary methods for TaqMan probes). Fold change in gene expression was reported as 2^−ΔΔCT^, where ΔΔC_T_ = *Phd2^f/f^;THCre* (C_T_target – C_T_reference) – *Phd2^f/f^* (C_T_target – C_T_reference).

### Statistical analysis

Data are shown as mean ± s.e.m. Statistical analyses were performed using unpaired Student’s *t*-tests, unless otherwise stated, and using GraphPad Prism 9.0 Software.

## Results

Since pseudohypoxic PPGLs have a characteristic gene expression profile, we first sought to test whether and to what extent this was mimicked by *Phd2* inactivation in the AM. To this end, we intercrossed mice bearing a conditionally inactivated (*Phd2^f/f^*) allele with a transgenic line expressing Cre recombinase restricted by the TH promoter (*THCre*) to generate *Phd2^f/f^;THCre* mice and measured effects on the expression of a panel of 14 genes that are frequently dysregulated in pseudohypoxic PPGLs (Supplementary Table 1). This comprised several classes of genes, including HIF target genes *Vegfa*, *Slc2a1* and *Ldha*; atypical mitochondrial subunits *Ndufa4l2* and *Cox4i2*; G-protein signalling pathway components *Rgs4*, *Rgs5* and *Adora2a*; *Pnmt*, the terminal enzyme in catecholamine synthesis; genes with oncogenic potential including *Stc1*.

**Figure 1:**
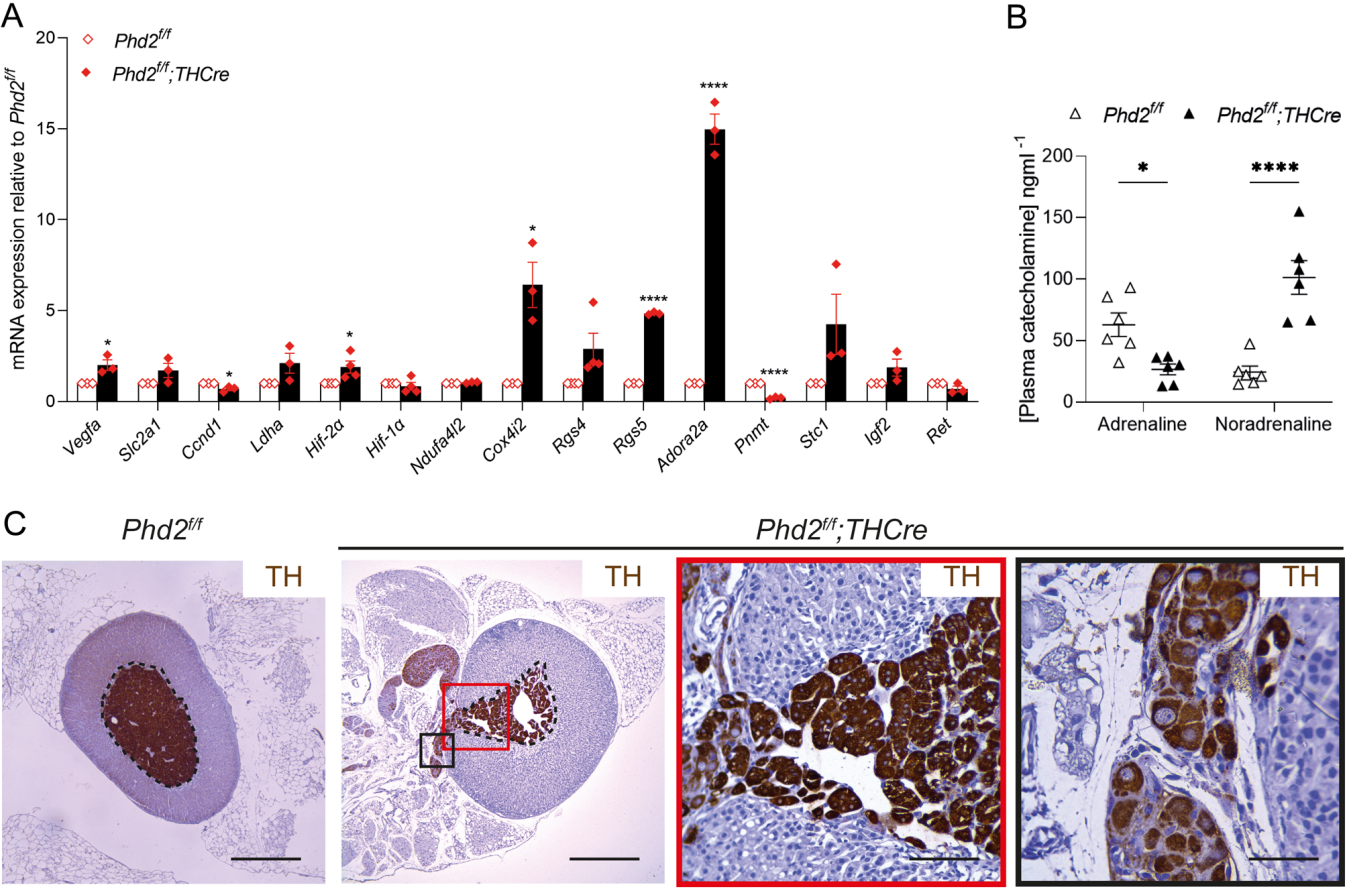
Effect of TH-restricted *Phd2* inactivation on gene expression, plasma catecholamines and AM morphology. (A) Gene expression profile of AMs in *Phd2^f/f^;THCre* mice (relative to *Phd2^f/f^* controls), analysed by RT-qPCR on RNA extracted from ten AMs per biological replicate. Data for individual genes were analysed by unpaired two-tailed Student’s *t*-tests. Gene expression pattern in *Phd2^f/f^;THCre* AMs resembles that of pseudohypoxic PPGLs. (B) Plasma catecholamine levels in *Phd2^f/f^;THCre* (vs *Phd2^f/f^* control) mice. Data were analysed by a two-way ANOVA, interaction (*Phd2* inactivation vs noradrenaline/adrenaline ratio) *P* < 0.0001 followed by Sidak’s multiple comparisons test. Graph shows a reduction in adrenaline and an increase in noradrenaline in the plasma of *Phd2^f/f^;THCre* mice. (C) Representative images of *Phd2^f/f^;THCre* (compared with *Phd2^f/f^* control) AGs. The AM is encircled with a black dashed line in this and other figures. TH antibody (brown); Harris haematoxylin counterstain (blue). Enlarged images show ectopic TH^+^ cells in the adrenal cortex (red frame) and in a peri-adrenal structure (black frame). Scale bars: 0.5 mm (far and middle left panels); 0.1 mm (red frame); 0.05 mm (black frame). Data show mean ± s.e.m. *P < 0.05, ****P < 0.0001.

These experiments revealed that many, though not all, genes identified as dysregulated in pseudohypoxic PPGLs were also dysregulated in a similar way in the AM of *Phd2^f/f^;THCre* mice (Fig. 1A), suggesting that their expression is directly or indirectly altered as a consequence of dysregulation of the PHD2/HIF system in this setting. Interestingly, not all these genes have been identified as being dynamically regulated by HIF itself in the manner observed. In particular, *Pnmt* has been reported to be upregulated by HIF-1 in rat pheochromocytoma PC12 cells (Tai* et al.* 2009), whereas we observed striking downregulation of *Pnmt* (mRNA and protein) with *Phd2* inactivation/HIF activation (Fig. 1A and Supplementary Fig. 1). PNMT is the terminal enzyme in the catecholamine synthesis pathway which converts noradrenaline into adrenaline. In keeping with the loss of *Pnmt*, we observed a shift in plasma catecholamines: an increase in noradrenaline together with a reduction in adrenaline (Fig. 1B). This predominantly noradrenergic secretory profile, together with a loss of *Pnmt* expression, is similar to clinical observations in patients with pseudohypoxic PCCs (Eisenhofer* et al.* 2001).

We therefore sought to determine whether *Phd2^f/f^*;*THCre* mice have morphological features of PCC, in particular evidence of intra-adrenal pathology. However, no frank tumours, nodules, cortical compression or loss of nest-like/formation of sheet-like chromaffin cell clusters (Smith-Hicks* et al.* 2000, Park* et al.* 2015) were observed, and there was no change in overall AM volume or proliferation (Supplementary Table 2).

However, we noted striking abnormalities of adrenal morphology, in particular the abnormal location of TH^+^ cells. These comprised clusters of ectopic cells in the adrenal cortex that disrupt the outer cortical boundary, adjacent to a TH^+^ extra-adrenal ganglion-like structure (Fig. 1C). Altogether, 11 out of 14 *Phd2^f/f^;THCre* mice and 0 out of 15 littermate controls manifest these abnormalities.

TH^+^ chromaffin cells migrate through the adrenal cortex to reach their final destination in the AM (Furlan* et al.* 2017, Hanemaaijer* et al.* 2021), during which time they acquire *Pnmt* expression in the final stages of maturation to become adrenergic (Verhofstad* et al.* 1979). We therefore considered whether the abnormally located TH^+^ cells (both within and directly adjacent to the AG) represent abnormal migration of chromaffin cells and that the reduction in *Pnmt* might also reflect failure to acquire *Pnmt* due to dysregulated/arrested development with *Phd2* inactivation.

**Figure 2:**
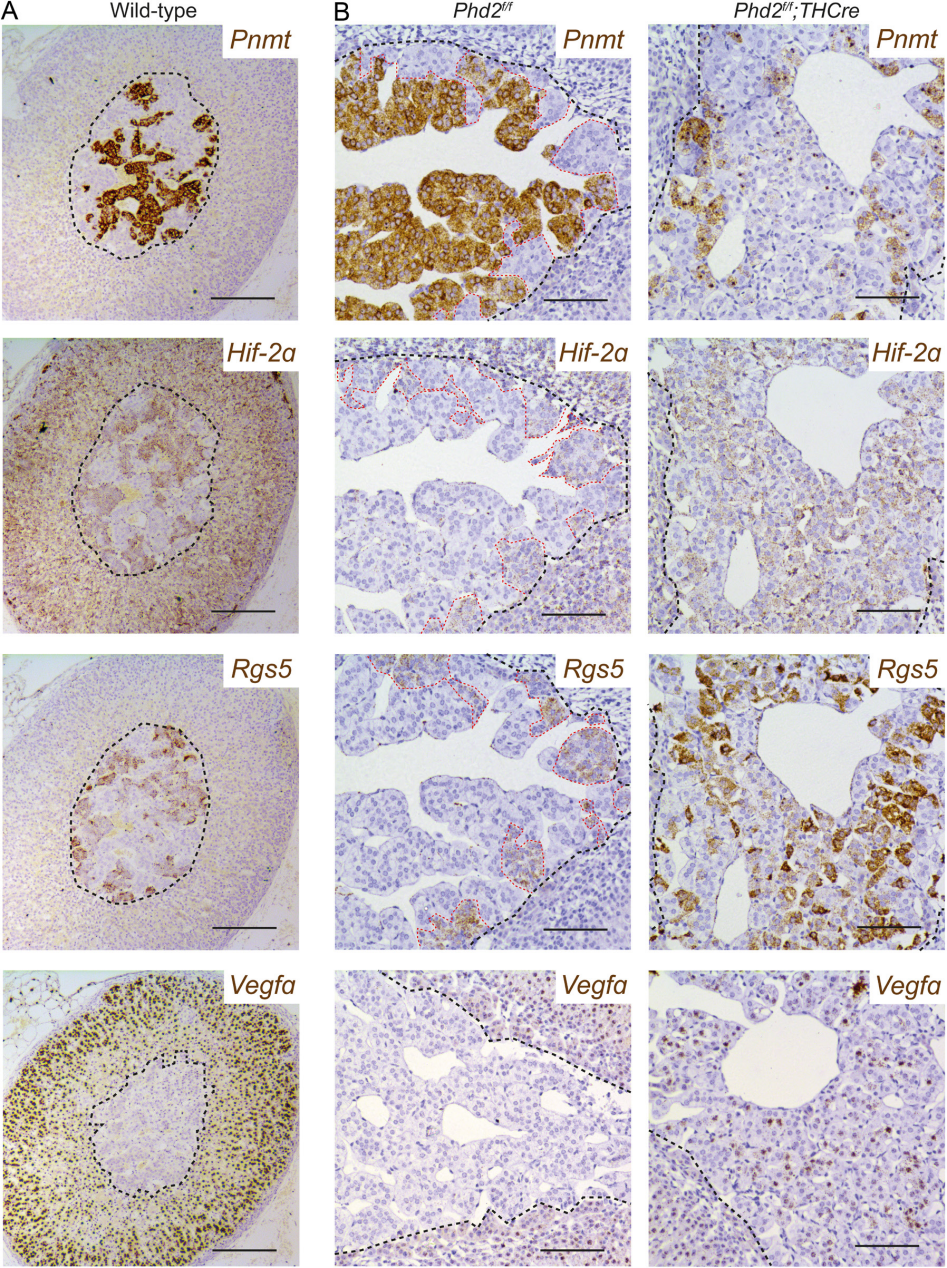
Effect of *Phd2* inactivation on the spatial expression of genes in the AM. (A) *In situ* hybridisation for *Pnmt*, *Hif-2α*, *Rgs5* and* Vegfa* mRNA (brown) in adjacent sections of a WT AG showing *Hif-2α* and *Rgs5* mRNA expression in *Pnmt*^−^ cells. Gill’s haematoxylin counterstain (grey-blue). Scale bars: 0.2 mm. (B) Comparison of *Pnmt*, *Hif-2α*, *Rgs5* and* Vegfa* mRNA expression in *Phd2^f/f^* vs *Phd2^f/f^;THCre* AMs. Red dashed lines outline *Pnmt*^−^/*Hif-2α*^+^/*Rgs5*^+^ cell populations in this and other figures. Images show a switch from predominantly *Pnmt*^+^/*Hif-2α*^−^/*Rgs5*^−^ to *Pnmt*^−^/*Hif-2α*^+^/*Rgs5*^+^ cells following *Phd2* inactivation. Harris haematoxylin counterstain (blue). Scale bars: 0.1 mm.

To address this, we proceeded to analyse the spatial distribution of gene expression within the AM. The normal AG is known to express *Pnmt* in a restricted set of mature chromaffin cells that produce adrenaline (Coupland & Hopwood 1966). Analysis of the spatial distribution of *Pnmt* expression in WT mice confirmed this, with the majority (~75%) of chromaffin cells expressing *Pnmt* (Fig. 2A). We next considered whether this expression pattern extended to other genes which were dysregulated in AMs with loss of *Phd2*. The following genes were selected for analysis: *Hif-2α*, since its upregulation is characteristic of VHL-associated neoplasia including PCCs (Toledo 2017); *Vegfa* and *Rgs5*, since these are reported HIF target genes (Jin* et al.* 2009, Fielding* et al.* 2018) and the latter is also a proposed regulator of chromaffin cell differentiation (Chan* et al.* 2019, Hanemaaijer* et al.* 2021). These experiments revealed a striking inverse pattern of gene expression, with *Hif-2α* and *Rgs5* mRNA being expressed only in the normal AM cells that do not express *Pnmt* (Fig. 2A). *Vegfa*, on the other hand, could not be detected, except in the adrenal cortex where it was strongly expressed (Fig. 2A). This suggested the possibility that a binary, cell-specific pattern of expression exists within AM cells, with low *Pnmt* associating with high *Hif-2α* and upregulation of at least some of its transcriptional targets such as *Rgs5*.

**Figure 3:**
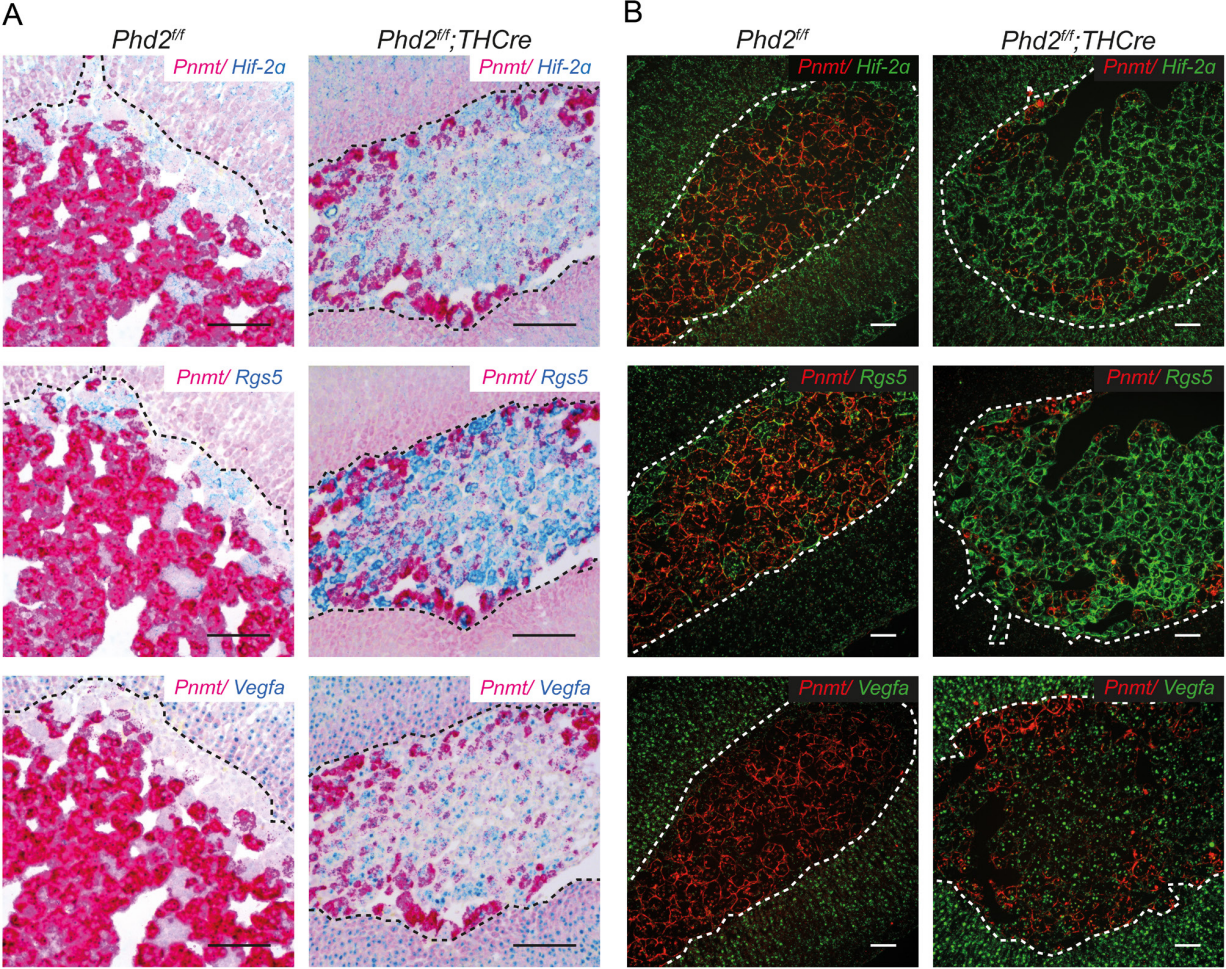
Spatial co-localisation of genes in the AM. (A) Dual *in situ* hybridisation or (B) proximity ligation *in situ* hybridisation (PLISH) for *Pnmt, Hif-2α, Rgs5* and* Vegfa* mRNA in *Phd2^f/f^* and *Phd2^f/f^;THCre* AMs. *Pnmt* expression in cells inversely correlates with *Hif-2α* and* Rgs5* in both genotypes but *Pnmt*^+^/*Hif-2α*^−^/*Rgs5*^−^ cell populations are dominant in *Phd2^f/f^* whereas this switches to *Pnmt*^−^/*Hif-2α*^+^/*Rgs5*^+^ cells in *Phd2^f/f^;THCre* AMs, which additionally show induction of *Vegfa*. For dual *in situ* hybridisation, Gill’s haematoxylin counterstain (grey-blue). Scale bars for dual *in situ* hybridisation: 0.1 mm, for PLISH: 0.05 mm.

The findings therefore raised the interesting question as to whether inactivation of *Phd2* might affect the expression of these genes (*Pnmt*, *Hif-2α*, *Rgs5* and *Vegfa*) within a specific population of cells or whether the prevalence of the two populations manifesting the observed patterns of gene expression might change after *Phd2* inactivation. To address this, we performed further *in situ* mRNA hybridisation studies. These studies revealed a major switch of cell populations in AMs following *Phd2* inactivation. The same pattern of *Pnmt*^−^/*Hif-2α*^+^/*Rgs5*^+^ was maintained, but cells manifesting this pattern now became the dominating cell population distributed across most of the AM with the exception of small areas around the periphery (Fig. 2B). Within the dominating population of *Pnmt*^−^/*Hif-2α*^+^/*Rgs5*^+^ expressing cells, there was also a mild induction of the HIF target gene *Vegfa* (Fig. 2B). This inverse expression of *Pnmt* and *Hif-2α*/*Rgs5*/*Vegfa* in *Phd2^f/f^* and *Phd2^f/f^;THCre* AMs was confirmed by dual *in situ* hybridisation and PLISH, which allow multiplexing of different mRNA probes to measure overlapping patterns of expression (Fig. 3 and Supplementary Fig. 2).

**Figure 4:**
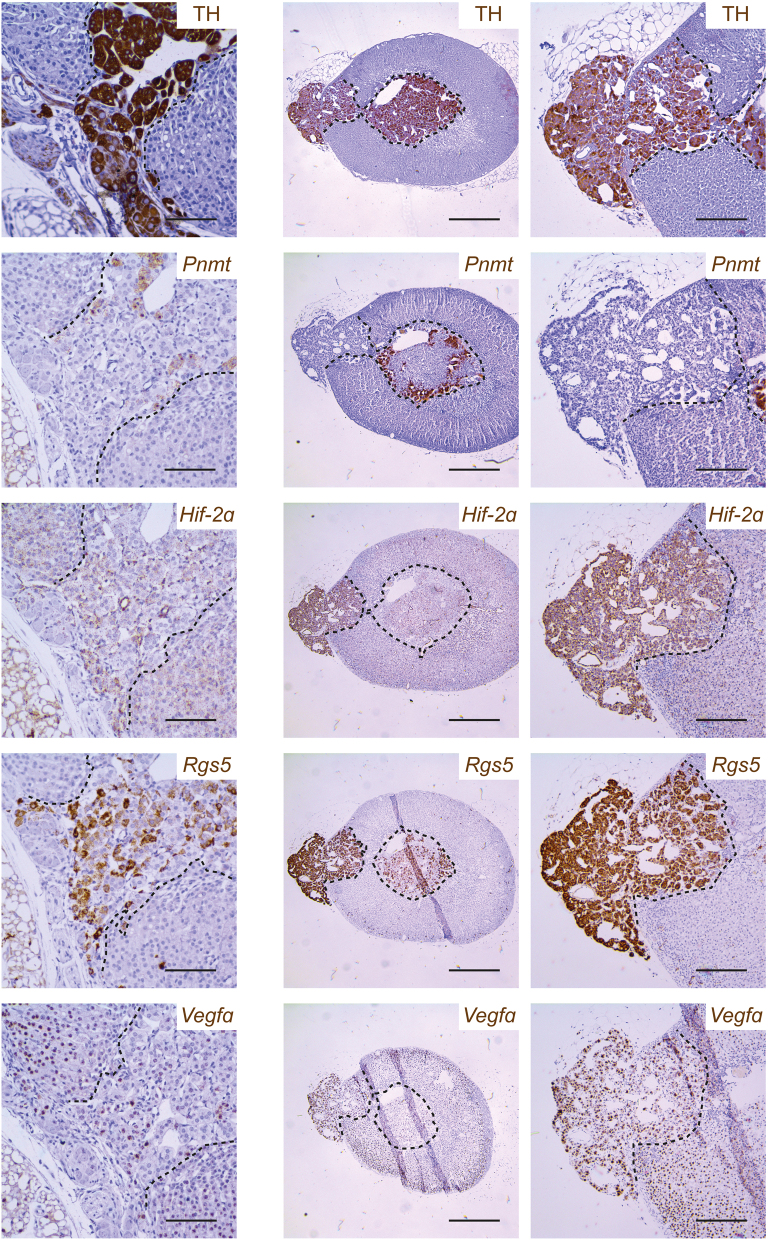
Gene expression profile of the ectopic TH^+^ cell tracks in adrenal glands from *Phd2^f/f^;THCre* mice. Representative images of ectopic TH^+^ cells in the adrenal cortex from two different adrenal glands (left vs middle and right hand columns) from *Phd2^f/f^;THCre* mice showing TH protein and *Pnmt*, *Hif*-*2α*, *Rgs5* and *Vegfa* mRNA. TH^+^ cells in these ectopic tracks are essentially all *Pnmt^−^*/*Hif-2α*^+^/*Rgs5*^+^/*Vegfa*^+^. Harris haematoxylin counterstain (blue). Scale bars: 0.05 mm (left column), 0.5 mm (middle column), 0.2 mm (right column).

We next went on to examine the ectopic TH^+^ cells within the adrenal cortex. Interestingly, essentially all these cells were *Pnmt*^−^/*Hif-2α*^+^/*Rgs5*^+^/*Vegfa*^+^ (Fig. 4) (and negative for the adrenal cortical cell marker *Cyp11a1*, Supplementary Fig. 3), again suggestive of arrested migration of an immature *Pnmt*^−^ population of chromaffin cells within the adrenal cortex during development. Taken together, these experiments suggest that TH-restricted *Phd2* inactivation results in a pattern of gene expression similar to that of pseudohypoxic PPGLs. Morphological abnormalities suggestive of an effect on adrenal development were coupled to a major switch in an apparently binary pattern of gene expression observed in populations of cells within the AM.

**Figure 5:**
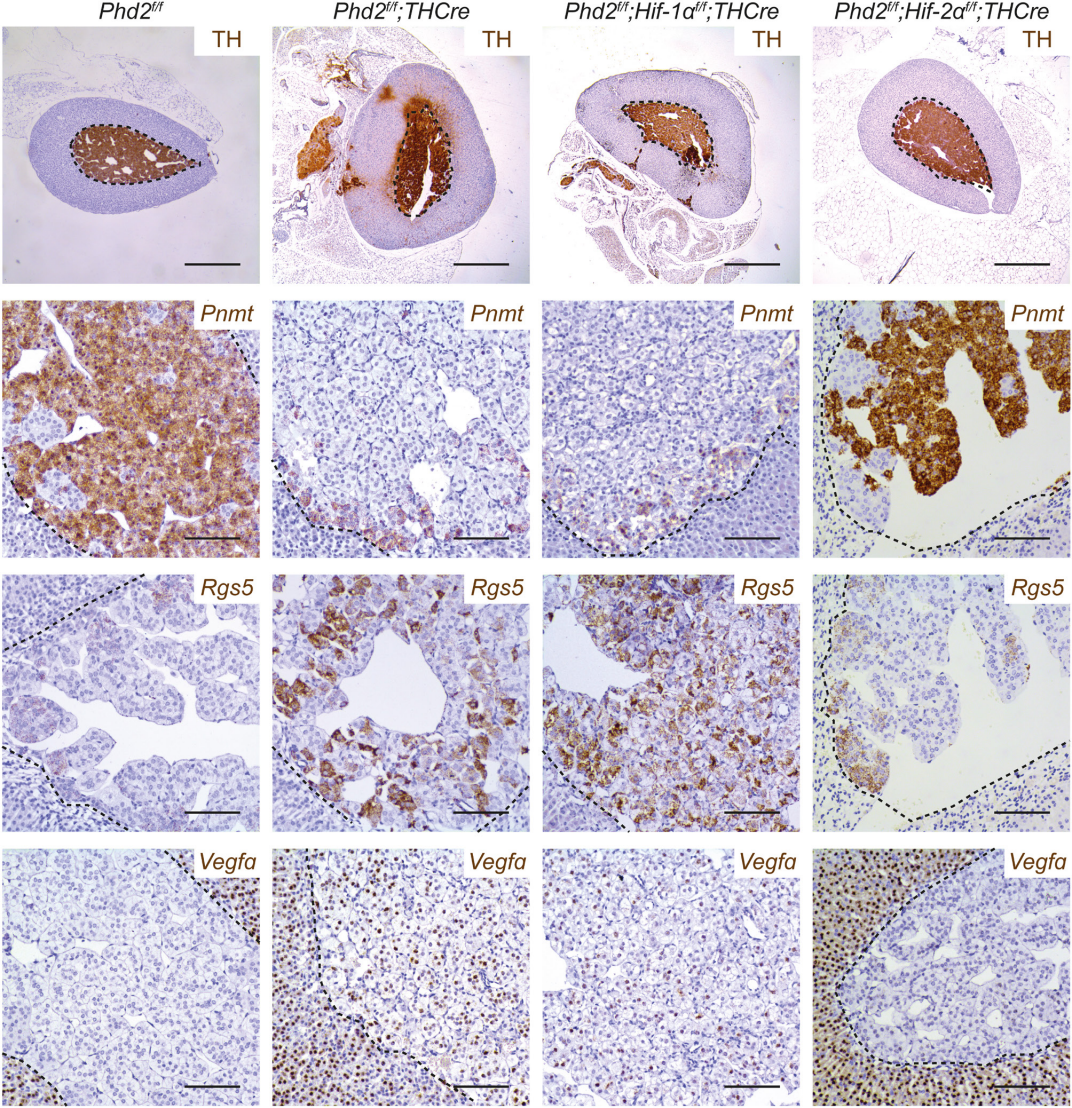
Effect of combined *Hif-α* and *Phd2* inactivation on morphological abnormalities and gene expression in the AM. Representative images of TH immunohistochemistry and *in situ* hybridisation for *Pnmt*,* Rgs5* and* Vegfa* mRNA in *Phd2^f/f^*, *Phd2^f/f^;THCre*, *Phd2^f/f^;Hif-1α^f/f^;THCre* and *Phd2^f/f^;Hif-2α^f/f^;THCre* AMs. Concomitant inactivation of *Hif-2α* and *Phd2* (but not *Hif-1α* and *Phd2*) reverses the morphological and gene expression changes observed in *Phd2^f/f^;THCre* mice such that these AMs resemble those of control (*Phd2^f/f^*) mice. Harris haematoxylin counterstain (blue). Scale bars: 0.5 mm (top row), 0.1 mm (lower panels).

To further understand this process, we intercrossed animals to generate *Phd2^f/f^;Hif-1α^f/f^;THCre* and *Phd2^f/f^*;*Hif-2α^f/f^;THCre* mice, which were examined with respect to the above phenotypes. No reversion of the morphological phenotype was observed with concomitant *Hif-1α* inactivation (Fig. 5). In striking contrast, *Hif-2α* inactivation (*Phd2^f/f^;Hif-2α^f/f^;THCre* mice) completely reversed all the morphological abnormalities associated with *Phd2* inactivation, such that AMs were similar to those of control (*Phd2^f/f^*) mice (Fig. 5). Similar results were obtained with analysis of *Pnmt*, *Rgs5* and *Vegfa* gene expression. The inverse expression pattern of *Pnmt* and *Rgs5* was invariant, but the proportion of cells of each type was strikingly different, with *Phd2^f/f^;Hif-1α^f/f^;THCre* mice retaining a dominant population of *Pnmt*^−^/*Rgs5*^+^ cells while *Phd2^f/f^;Hif-2α^f/f^;THCre* mice apparently reverted to a phenotype indistinguishable from controls, including loss of *Vegfa* mRNA (Fig. 5). Together, this indicates that *Hif-2α*, not *Hif-1α*, is necessary for the abnormal phenotype resembling pseudohypoxic PCCs.

We therefore hypothesised that inactivation of *Phd2* might have at least two distinct effects in order to generate this pseudohypoxic phenotype: first, a dynamic induction of HIF transcriptional target genes including *Vegfa*; secondly, a switch to an immature noradrenergic cellular phenotype within the AM. Since we also observed morphological abnormalities suggestive of interrupted differentiation and/or migration, we sought to determine whether these components of the pseudohypoxic phenotype might reflect an action of the PHD2/HIF-2 axis during differentiation. To test this, we proceeded to compare the effects with those in animals where inactivation of *Phd2* was restricted to adult life, using two different models. We analysed adult *Phd2^f/f^;THCreER* at 3 months after inducing recombination with tamoxifen. To assess any effect of more extensive recombination, adult *Phd2^f/f^;RosaCreER* mice were also studied. Since these develop systemic abnormalities (Hodson* et al.* 2016), they were studied somewhat earlier, approximately 17 days after tamoxifen dosing.

**Figure 6:**
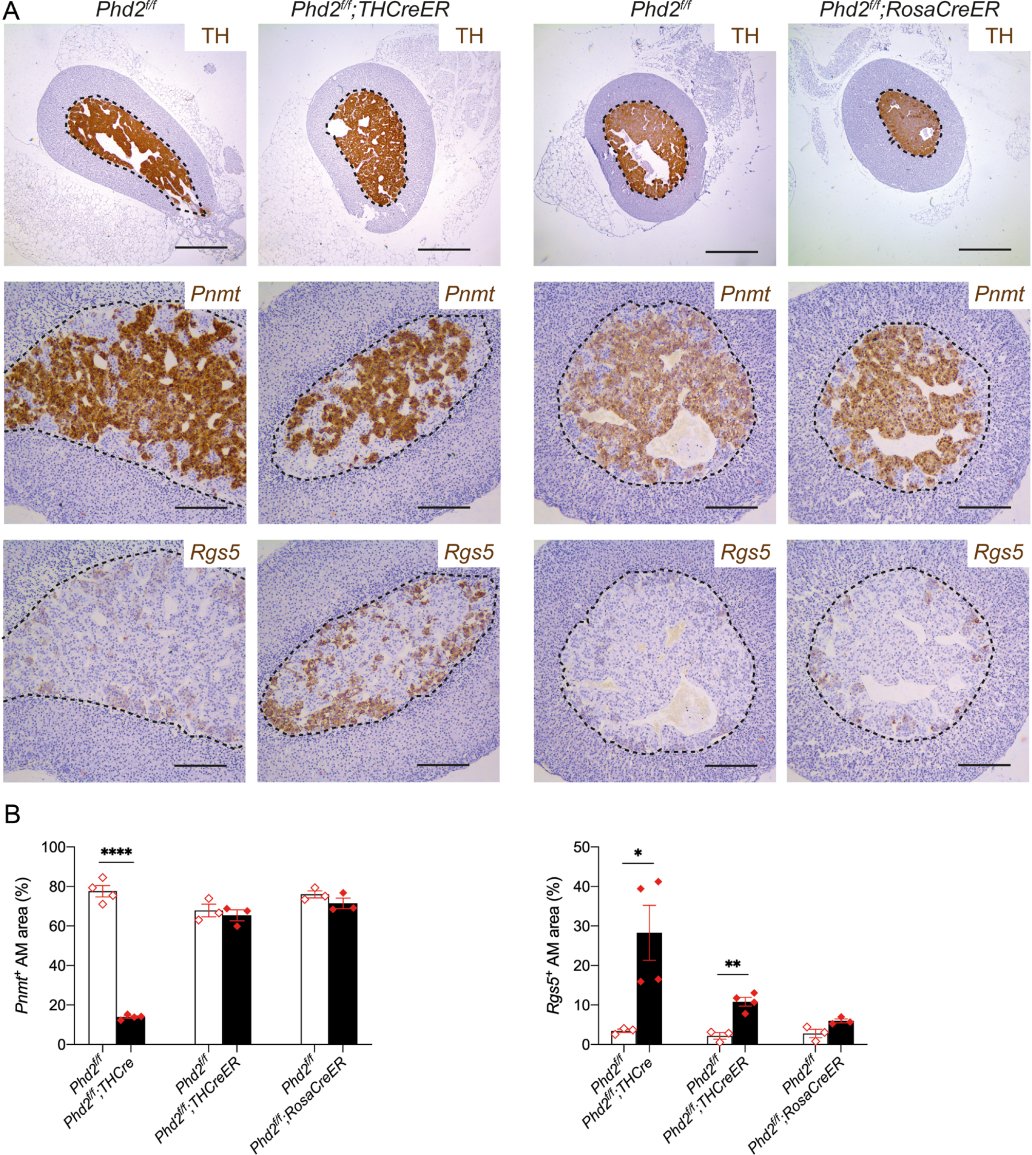
Effect of adult-onset *Phd2* inactivation on morphology and gene expression in the AM. (A) Representative images of TH protein and *Pnmt* and *Rgs5* mRNA in adrenal glands from *Phd2^f/f^;THCreER*, *Phd2^f/f^;RosaCreER* (and their respective *Phd2^f/f^* controls) killed ~3 months or 17 days post tamoxifen treatment, respectively. Harris haematoxylin counterstain (blue). Scale bars: 0.5 mm (top row) or 0.2 mm (bottom two rows). (B) Quantification of the percentage of *Pnmt*^+^ and *Rgs5*^+^ AM area; AMs from *Phd2^f/f^;THCre*, *Phd2^f/f^;THCreER* and *Phd2^f/f^;RosaCreER* mice (filled bars and rhombi) and their respective controls (open bars and rhombi). Bars show mean ± s.e.m. Data within individual genotype groups were compared by unpaired two-tailed Student’s *t*-tests: *P < 0.05, **P < 0.01, ****P < 0.0001. Adult-onset *Phd2* inactivation did not phenocopy morphological abnormalities or *Pnmt* loss in the AM observed with early-onset *Phd2* inactivation, although a small but significant induction in *Rgs5*^+^ was noted within the *Pnmt^−^* cells of *Phd2^f/f^;THCreER* AMs.

**Figure 7:**
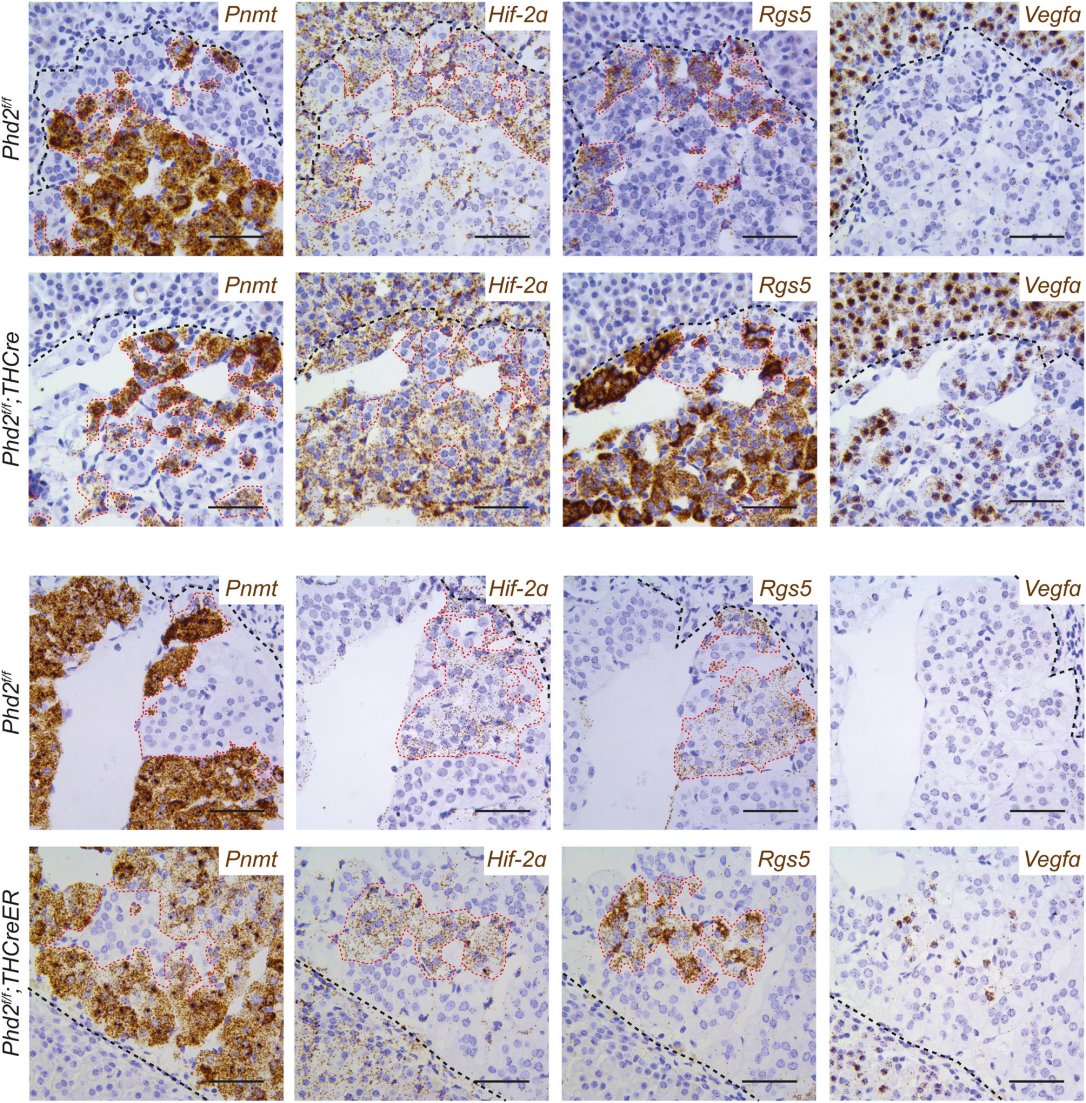
Spatial distribution of gene expression in the AM with constitutive or adult-onset *Phd2* inactivation. Representative images of *in situ* hybridisation for *Pnmt*, *Hif-2α*, *Rgs5* and *Vegfa* mRNA in *Phd2^f/f^;THCre* (constitutive) or *Phd2^f/f^;THCreER* (adult-onset) mice and their respective controls. *Pnmt* expression in cells inversely correlates with *Hif-2α* and* Rgs5* across all genotypes but the *Pnmt*^+^/*Hif-2α*^−^/*Rgs5*^−^ cell population is dominant in *Phd2^f/f^* controls whereas this switches to *Pnmt*^−^/*Hif-2α*^+^/*Rgs5*^+^ cells in *Phd2^f/f^;THCre* AMs. This contrasts to *Phd2^f/f^;THCreER* AMs, where *Pnmt*^+^/*Hif-2α*^−^/*Rgs5*^−^ cells remain the dominant population; additionally, *Rgs5* and *Vegfa* are induced within the minority population of *Pnmt*^−^/*Hif-2α*^+^/*Rgs5*^+^ cells compared to control mice. Harris haematoxylin counterstain (blue). Scale bars: 0.05 mm.

In striking contrast to *Phd2^f/f^;THCre* mice, inactivation of *Phd2* in adult mice by either protocol did not result in morphological abnormalities or a change in the spatial distribution of *Pnmt*^−^ and *Pnmt*^+^ cells (Fig. 6). Additionally, no change in AM volume or proliferation was noted (Supplementary Table 2). In contrast, induction of *Rgs5* and *Vegfa* mRNA was observed in both models of adult-onset *Phd2* inactivation and was apparently confined to the population of cells that did not express *Pnmt* and were *Hif-2α*^+^ (Figs 6, 7 and Supplementary Fig. 4). Together, this suggests that two distinct effects of HIF-2 activation contribute to the pseudohypoxic phenotype observed with TH-restricted *Phd2* inactivation: first, as a regulator of chromaffin cell differentiation during development; secondly, as a dynamic regulator of HIF target gene expression within *Hif-2α* expressing cells.

## Discussion

Our findings demonstrate that TH-restricted inactivation of *Phd2* results in a pattern of gene expression within the AM that resembles pseudohypoxic PPGLs. Importantly, several lines of evidence reveal that in addition to dynamic activation of the HIF transcriptional response, this alteration in gene expression reflects developmental consequences of* Phd2* inactivation on the AM. First, the alteration in gene expression involves spatial changes in cell-specific patterns of gene expression that reflect lack of terminal differentiation to *Pnmt*^+^ cells. Secondly, several of the genes involved in the altered pattern of expression, including *Pnmt* and *Hif-2α* itself, are not dynamic HIF transcriptional targets. Thirdly, altered patterns of gene expression were associated with morphological abnormalities, including ectopic TH^+^ cell populations with a *Pnmt*^−^ pattern of gene expression. Finally, neither the spatial change in gene expression within the AM nor the morphological abnormalities in the position of TH^+^ cells could be induced by *Phd2* inactivation in adult life.

These findings are of particular interest when considered alongside several unusual observations on the clinical genetics of PPGL. Mutations that directly affect components of the PHD-HIF system are frequently observed in the uncommon syndrome of pseudohypoxic PPGL, but rarely, if ever, seen in other much more common forms of cancer. This is surprising since dynamic regulation of the transcriptional response to hypoxia by the PHD-HIF system is a general function observed in all cells. In addition, pseudohypoxic PPGL is associated with a very much higher incidence of germline (or post-zygotic but early somatic) mutations vs sporadic mutations, as compared to other forms of neoplasia.

Our findings suggest a mechanism by which these apparently paradoxical findings could be explained. Specifically, the observation that dysregulation of the PHD-HIF system has a tissue-specific action on AM development suggests that these tumours have origins in early life, with PHD2-dependent cell differentiation changes predisposing to subsequent tumourigenesis. Interestingly, inactivation of the related HIF prolyl hydroxylase isoform PHD3 prevents developmental culling of neurons, and this has also been proposed to pre-dispose to tumours (Lee* et al.* 2005, Bishop* et al.* 2008). Consistent with the relevance of our findings to the human syndrome, we found that changes in adrenal morphology and gene expression were ablated by inactivation of *Hif-2α,* but not *Hif-1α*, in line with the observation that activating mutations in *HIF-2α*, but not *HIF-1α*, are associated with human PPGL (Buffet* et al.* 2020). The finding of extra-adrenal tissue reported in our study might also be relevant to the not infrequent occurrence of chromaffin cell tumours at extra-adrenal sites (Tischler 2008). Although a correlation between the distribution of chromaffin tissue and paraganglioma has been reported (Coupland 1965), our data raises the possibility that these extra-adrenal PGLs may arise due to impaired migration and differentiation of sympathetic precursors that would normally populate the adrenal primordia to acquire features of mature, adrenergic chromaffin cells during development (Furlan* et al.* 2017, Hanemaaijer* et al.* 2021).

The precise action of HIF-2 in promoting abnormal adrenal development will require further investigation, including the dissection of effects on differentiation, migration and interaction with other processes including innervation (Vollmer 1996). Notably, inactivation of *Hif-2α* in the setting of *Phd2* inactivation (Supplementary Fig. 5) did not obliterate *Pnmt*^−^/*Rgs5*^+^ cells in *Phd2^f/f^;Hif-2α^f/f^*;*THCre* mice. Furthermore, in the normal AM, *Pnmt*^−^ cells that show increased *Hif-2α* and *Rgs5* mRNA levels did not express detectable levels of HIF-2α protein (data not shown). Thus, in this cell population, HIF-2 does not appear necessary to generate a *Pnmt*^−^/*Hif-2α*^+^/*Rgs5*^+^ or noradrenergic phenotype; nor is HIF-2 necessary for PNMT acquisition in adrenergic cells, as evidenced from the normal AMs in *Hif-2α^f/f^;THCre* mice (Macias* et al.* 2018) as well as in *Phd2^f/f^;Hif-2α^f/f^;THCre* mice reported here. Rather, excess stabilisation of HIF-2α acts in some way to interrupt a developmental programme that ordinarily generates *Pnmt*^+^ in other AM cells.

Although *Phd2* inactivation resulted in changes characteristic of pseudohypoxic PCCs, in this model we did not detect frank PCCs in ~3 month-old mice. Since PCC development might have a longer latency, we also analysed older cohorts of animals but again did not observe PCC development in *n*  = 7 *Phd2^f/f^;THCre* and littermate control mice analysed aged ~18 months. However, we did observe three TH^+^, chromogranin A^+^ nodules of chromaffin cells amongst a group of *n*  = 11 *Phd2^f/f^;Hif-1α^f/f^;THCre* mice aged in parallel (Supplementary Fig. 6). The significance of this is unclear, but HIF-1α has been reported to act as a tumour suppressor in VHL-associated renal clear cell carcinoma (Shen* et al.* 2011), and it may be that HIF-1 also has a tumour suppressive role in this context.

Our findings have relevance for both experimental and clinical research into PCC. They suggest that it may also be useful to revisit mouse models of pseudohypoxic PCC including models of inactivation of *Sdhx* that have not resulted in PCC (Piruat* et al.* 2004, Bayley* et al.* 2009, Díaz-Castro* et al.* 2012, Macias* et al.* 2014, Lepoutre-Lussey* et al.* 2016, Al Khazal* et al.* 2021), to assess whether a switch from *Pnmt*^+^/*Hif-2α*^−^/*Rgs5*^−^ to *Pnmt*^−^/*Hif-2α*^+^/*Rgs5*^+^ cell populations also occurs in these settings. At the clinical level, our findings suggest that attempts at prevention or treatment should rationally include a focus on early life. Interestingly, patients with congenital cyanotic heart disease (a condition associated with life-long hypoxaemia beginning perinatally) have been reported to be susceptible to PCCs, many of which harbour sporadic *HIF-2α* mutations (Opotowsky* et al.* 2015, Vaidya* et al.* 2018); our data suggests that it is the early-onset hypoxia in these patients which predisposes to subsequent PCC formation.

Interestingly, the HIF-2-dependent effects of *Phd2* inactivation, including paraganglioma-like carotid bodies (Fielding* et al.* 2018), are strikingly different from those of *Vhl* inactivation in catecholaminergic tissues using the same *THCre* promoter as in this study, which results in atrophy of multiple organs of the sympathetic nervous system, including the AM and the carotid body (Macias* et al.* 2014). There is a complex association between mutations in VHL disease and the tumour phenotypes, with type 2 *VHL* mutations that result in PCCs having only modest (or minimal) effects on HIF dysregulation, while type 1 mutations do not develop PCCs and result in greater HIF stabilisation (Kaelin 2008). Although levels of HIF activation have not been compared directly, our findings support the hypothesis that more moderate HIF activation associated with inactivation of a single *PHD* (as opposed to another function of VHL distinct from an action on HIF) is the most likely explanation for this paradox.

Several other aspects of the dysregulated gene expression pattern merit comment. In particular, several genes that were upregulated in *Phd2*-inactivated AMs (including *Rgs5*, *Cox4i2* and *Adora2a*) are very highly and specifically expressed in normal carotid body type I cells (Zhou* et al.* 2016), a cell type that responds to low oxygen with the rapid release of neurotransmitters to mediate hypoxic ventilatory control in what is termed acute oxygen sensing (Gao* et al.* 2019). Chromaffin cells are also reportedly acutely oxygen-sensitive during development, but this is lost in adulthood (Thompson* et al.* 1997). The retention of an immature phenotype in AMs with *Phd2* inactivation may extend beyond gene expression to include retention of acute oxygen sensitivity. In future studies, it will be of interest to determine the extent to which *Phd2* inactivation in the AM recreates oxygen sensitivity in the adult.

## Supplementary Material

Supplementary Materials

Supplementary Table 1. Genes commonly dysregulated in pseudohypoxic PPGLs. Genes from several classes (HIF target genes, G protein signalling pathway components, atypical mitochondrial subunits and genes with oncogenic potential) which are commonly dysregulated in pseudohypoxic PPGLs were identified from human studies, as detailed below.

Supplementary Table 2. Volumes and proliferation of AMs with constitutive and adult-onset Phd2 inactivation. Quantification of AM volumes and Ki67+ cells per mm2 of AMs in Phd2f/f;THCre, Phd2f/f;THCreER, Phd2f/f;RosaCreER mice and their littermate controls (Phd2f/f). Data were analysed by Student’s two tailed t-tests and are shown as mean±SEM; n=3 for all comparisons except volume in Phd2f/f;THCre versus control, where n=5. No significant differences in AM volume or proliferation were noted in pairwise comparisons between Phd2 knock-out versus control mice in any of the groups, although there were differences in values (including controls) between the paired comparisons which may be due to small differences in methods of tissue preparation.

Supplementary Figure 1. Effect of Phd2 inactivation on PNMT protein in AMs. PNMT immunohistochemistry (brown) in the AMs of Phd2f/f and Phd2f/f;THCre mice. AMs are outlined by a black dashed line in this and other figures. Images show loss of PNMT protein after Phd2 inactivation. Harris hematoxylin counterstain (blue). Scale bars: 0.1mm.

Supplementary Figure 2. Effects of TH-restricted Phd2 inactivation on spatial expression of genes in the AM. Representative images of in situ hybridisation for Hif-2α (pink) together with Pnmt, Rgs5 or Vegfa (blue) mRNA in AMs of Phd2f/f and Phd2f/f;THCre mice. Hif-2α expression in cells inversely correlates with Pnmt, but coincides with Rgs5, in both genotypes. Pnmt+/Hif-2α-/Rgs5- cell populations are dominant in Phd2f/f whereas this switches to Pnmt-/Hif-2α+/Rgs5+ cells in Phd2f/f;THCre AMs, which additionally show induction of Vegfa. Gill’s hematoxylin counterstain (grey-blue). Scale bars: 0.1mm.

Supplementary Figure 3. Adrenal cortical marker expression in adrenal glands of Phd2f/f;THCre mice. In situ detection of the adrenal cortical marker Cyp11a1 (red), Th (green, top panels), Pnmt (white, bottom panels) and Rgs5 (green, bottom panels) in the adrenal glands of Phd2f/f;THCre mice with bottom, left hand panel showing the adrenal medulla and the other panels the ectopic Th+ cell tracks. DAPI (blue) serves as a nuclear marker. Th+ cells are outlined by a white dashed line. Ectopic Th+ cells in the adrenal cortex, like Th+ cells in the AM, do not express Cyp11a1 mRNA but express high levels of Rgs5 and minimal Pnmt. Scale bars: 0.1mm (top left panel), 0.05mm (top right and bottom panels).

Supplementary Figure 4. Spatial distribution of gene expression in the AM with ubiquitous, adult-onset Phd2 inactivation. Representative images of in situ hybridisation for Pnmt, Hif-2α, Rgs5 and Vegfa mRNA in the AMs of Phd2f/f;RosaCreER mice and their littermate controls. Pnmt-/Hif-2α+/Rgs5+ area is outlined by a red dashed line in this and other figures. Pnmt expression in cells inversely correlates with that of Hif-2α and Rgs5 and Pnmt+/Hif-2α-/Rgs5- cells are the dominant population in both control Phd2f/f and Phd2f/f;RosaCreER mice. However, Rgs5 and Vegfa are additionally induced within the minority population of Pnmt-/Hif-2α+/Rgs5+ cells in Phd2f/f;RosaCreER compared to those in control Phd2f/f mice. Harris hematoxylin counterstain (blue). Scale bars: 0.05mm.

Supplementary Figure 5. Hif-1/2α expression in the AMs of mice with concomitant Phd2 and Hif-1/2α inactivation. Representative images of in situ hybridisation for Pnmt, Hif-2α and Hif-1α in the AMs of Phd2f/f, Phd2f/f;THCre, Phd2f/f;Hif-1αf/f;THCre and Phd2f/f;Hif-2αf/f;THCre mice. Loss of Hif-2α mRNA in Phd2f/f;Hif-2αf/f;THCre compared to mice of other genotypes (using a customised Hif-2α mRNA probe which targets the floxed exon 2 in Phd2f/f;Hif-2αf/f;THCre mice). Hif-1α mRNA is barely detectable in AMs of any genotype. Counterstain for Pnmt and Hif-1α: Harris hematoxylin (blue); for Hif-2α: Gill’s hematoxylin (grey-blue). Scale bars: 0.1mm (top two rows) and 0.05mm (bottom two rows).

Supplementary Figure 6. TH+ nodule in the AM of an aged mouse with concomitant inactivation of Phd2 and Hif-1α. TH and Ki67 immunohistochemistry in the AMs of ~18 month-old Phd2f/f;Hif-1αf/f;THCre and littermate control (Phd2f/f) mice. Higher magnification images denoted with: a black box in the control animal; red boxes to show the nodule in the Phd2f/f;Hif-1αf/f;THCre mouse. Red arrowheads indicate Ki67+ nuclei, which are more frequent within the nodule compared to surrounding AM or AM of control mouse. Harris hematoxylin counterstain (blue). Scale bars: 0.5mm (low magnification TH images), 0.1mm (higher magnification TH image and lower magnification Ki67 images) and 0.05mm (higher magnification Ki67 image).

## Declaration of interest

P J R is a scientific co-founder of, and holds equity in, ReOx Ltd, a University spin-out company that seeks to develop therapeutic HIF hydroxylases inhibitors and a non-executive director of Immunocore Holdings PLC. E J H is employed under the Cambridge Experimental Medicine Initiative, partly funded by AstraZeneca, although they have not been involved in this project. The other authors declare no financial interests.

## Funding

Funding for the work was received from the Oxford Branch of the Ludwig Institute for Cancer Research, the Wellcome Trust (106241/Z/14/Z) and the Paradifference Foundation. This work was also supported by the Francis Crick Institute, which receives its core funding from Cancer Research UK (FC001501), the UK Medical Research Council (FC001501), and the Wellcome Trust (FC001501). L E was sponsored by an MD fellowship from Boehringer Ingelheim Fonds; J D C C L by a FAPESP fellowship (2018/20083-1); S K by a Christopher Welch Scholarship and the Clarendon Fund.

## Author contribution statement

Experiments were designed by L E, M P B, P J R and T B. Data were collected and analysed by all authors. Manuscript was prepared by L E, M P B, P J R and T B and reviewed by all authors. Figures were prepared and statistical analyses performed by L E and M P B with input from other authors. P J R and T B conceived the study and managed the project. P J R and T B are co-senior authors.
