# Supplementary material for: Developmental role of PHD2 in the pathogenesis of pseudohypoxic pheochromocytoma
Source: Endocr Relat Cancer. 2021 Sep 20;28(12):757–72. doi: 10.1530/ERC-21-0211 (PMC8558849; doi:10.1530/ERC-21-0211)
Supplement: Supplementary Materials [file supplementary_material.pdf]

Supplementary Information

Developmental role of PHD2 in the pathogenesis of pseudohypoxic pheochromocytoma

Luise Eckardt[1,2], Maria Prange-Barczynska[1,3], Emma J. Hodson[4,5], James W. Fielding[1,3], Xiaotong Cheng[1,3], Joanna D. C. C. Lima[1], Samvid Kurlekar[1], Gillian Douglas[6], Peter J. Ratcliffe[1,3,4] and Tammie Bishop[1]

[1]Target Discovery Institute, University of Oxford, Oxford, UK

[2]Institute of Physiology and Pathophysiology, University of Heidelberg, Heidelberg, Germany

[3]Ludwig Institute for Cancer Research, University of Oxford, Oxford, UK

[4]The Francis Crick Institute, London, UK

[5]The Department of Experimental Medicine and Immunotherapeutics, University of Cambridge

[6]BHF Centre of Research Excellence, Division of Cardiovascular Medicine, Radcliffe Department of Medicine, John Radcliffe Hospital, University of Oxford, Oxford, UK

Authorship note: LE and MPB are joint first authors. PJR and TB are co-senior authors.

Supplementary Methods

RT-qPCR. The following TaqMan gene expression assays (Thermo Fisher Scientific, Waltham, US) were used to detect mRNA in Figure 1A: *Vegfa* (Mm00437306_m1), *Slc2a1* (Mm00441480_m1), *Ccnd1* (Mm00432359_m1), *Ldha* (Mm01612132_g1), *Epas1* (Mm00438712_m1), *Hif1a* (Mm00468869), *Ndufa4l2* (Mm01160374_g1), *Cox4i2* (Mm00446387_m1), *Rgs4* (Mm00501389_m1), *Rgs5* (Mm00501393_m1), *Adora2a* (Mm00802075_m1), *Pnmt* (Mm00476993_m1), *Stc1* (Mm01322191_m1), *Igf2* (Mm00439564_m1), *Ret* (Mm00436304_m1), *Actb* (Mm01205647_g1).

Immunohistochemistry. PNMT and Ki67 immunohistochemistry in Supplementary Table 2 and Supplementary Figures 1 and 6 was performed as described in the main Methods, using the following rabbit polyclonal antibodies: anti-PNMT (1:500, Enzo Life Science, Exeter, UK) and anti-Ki67 (1:5000, NB110-89719, Novus Biologicals, Cambridge, UK).

In situ hybridisation. Hif-2α mRNA in the AMs was tested by: in Supplementary Figure 2: dual *in situ* hybridisation with a Mm-Epas1 probe (314371-C2); in Supplementary Figure 5: using the BaseScope Reagent Kit v2 – RED (Advanced Cell Diagnostics, Newark, US), with a custom-designed probe targeted to the floxed exon 2 of mouse *Hif-2α* (as described in Fielding *et al.*, 2018).

Proximity Ligation in situ hybridisation. The following oligonucleotides were designed to detect mRNA of target genes in Figure 3B and Supplementary Figure 3.

Hif-2α
HLC2-VB2-mHif-2α-100: AGGTCAGGAATACTTAGCTATTGATGGTGGAGTTCCGTTTAGCCCCG
HRC2-VB2-mHif-2α-100: GAGAGCAGCGACAAGGGACCTTCTGTGTAGACGACTATAGCCAGGTT
HLC2-VB2-mHif-2α-291: AGGTCAGGAATACTTAGCTATTGATGGTCGCAGGTCCGAGAGTCCC
HRC2-VB2-mHif-2α-291: GTGTGAAAGGTCTTTAGTGCTTCTGTGTAGACGACTATAGCCAGGTT
HLC2-VB2-mHif-2α-1492: AGGTCAGGAATACTTAGCTATTGATGGTGGTCCATGGAGAACACC
HRC2-VB2-mHif-2α-1492: GCTTGAACAGGGATTCGGTCTTCTGTGTAGACGACTATAGCCAGGTT
HLC2-VB2-mHif-2α-4086: AGGTCAGGAATACTTAGCTATTGATGGTTTTAGATATTACAGAGTAC
HRC2-VB2-mHif-2α-4086: GCAAGATCCCATTTCTAAGCTTCTGTGTAGACGACTATAGCCAGGTT
HLC2-VB2-mHif-2α-4344: AGGTCAGGAATACTTAGCTATTGATGGTGAGAACACGAAAAGATAGC
HRC2-VB2-mHif-2α-4344: CACACACGTACATAATACACTTCTGTGTAGACGACTATAGCCAGGTT
HLC2-VB2-mHif-2α-5092: AGGTCAGGAATACTTAGCTATTGATGGTTCAAGTTCCGTCGCCCGCC
HRC2-VB2-mHif-2α-5092: CATTTGCATGTCAATAACCCTTCTGTGTAGACGACTATAGCCAGGTT
HLC2-VB2-mHif-2α-5177: AGGTCAGGAATACTTAGCTATTGATGGTCAGAGTGTCTTTAGTAGAA
HRC2-VB2-mHif-2α-5177: TACGAAACTCGGAATATTTCTTCTGTGTAGACGACTATAGCCAGGTT

HLC2-VB2-mHif-2α-5291: AGGTCAGGAATACTTAGCTATTGATGGTCCCGTACAGTTATAATGTC<br>HRC2-VB2-mHif-2α-5291: ATTTCCTTATAAAGTTAAGCTTCTGTGTAGACGACTATAGCCAGGTT
Pnmt
HLC2-VB3-mPnmt-43: AGGTCAGGAATACCAGGTTGTAATGGTGTAGCGTGCTTCAGGTCTG<br>HRC2-VB3-mPnmt-43: TTCGGGTCTGAGCCACTCCCTTAGTATGATGACAATATAGCCAGGTT<br>HLC2-VB3-mPnmt-101: AGGTCAGGAATACCAGGTTGTAATGGTGGCCAGCGTCGGAGTCAG<br>HRC2-VB3-mPnmt-101: AAGCCAAGGCGACAGCTACCTTAGTATGATGACAATATAGCCAGGTT<br>HLC2-VB3-mPnmt-134: AGGTCAGGAATACCAGGTTGTAATGGTCGAAGCGCTGGTAAGCCAA<br>HRC2-VB3-mPnmt-134: CGGAGATAGGCGCGGGGCTTAGTATGATGACAATATAGCCAGGTT<br>HLC2-VB3-mPnmt-443: AGGTCAGGAATACCAGGTTGTAATGGTCAATGAGGCAGGCATGCTG<br>HRC2-VB3-mPnmt-443: GCCAGGACTCACCCTTGTCCTTAGTATGATGACAATATAGCCAGGTT<br>HLC2-VB3-mPnmt-501: AGGTCAGGAATACCAGGTTGTAATGGTCGCTTCACCCTCGCTCGAA<br>HRC2-VB3-mPnmt-501: TGCACATCGATAGGCAGGACTTAGTATGATGACAATATAGCCAGGTT<br>HLC2-VB3-mPnmt-625: AGGTCAGGAATACCAGGTTGTAATGGTGGAAGCTAGTAAGATCTGG<br>HRC2-VB3-mPnmt-625: TGATGTGATGCAAAGCCCGCTTAGTATGATGACAATATAGCCAGGTT<br>HLC2-VB3-mPnmt-788: AGGTCAGGAATACCAGGTTGTAATGGTCGTAACCACCAAGGACCAG<br>HRC2-VB3-mPnmt-788: GGTGCGAAGCTCTCGGACCTTAGTATGATGACAATATAGCCAGGTT
Rgs5
HLC2-VB1-mRgs5-28: AGGTCAGGAATACTTACGTCGTTATGGTGACCGTCTGTGGAAACTTC<br>HRC2-VB1-mRgs5-28: AGCACGAACCTCTCAACAGCTTATAGGTCGAGTAGTATAGCCAGGTT<br>HLC2-VB1-mRgs5-265: AGGTCAGGAATACTTACGTCGTTATGGTGGCGCCACTGCAGGACCT<br>HRC2-VB1-mRgs5-265: GGAGAAGCTTGTCCAGGGACTTATAGGTCGAGTAGTATAGCCAGGTT<br>HLC2-VB1-mRgs5-485: AGGTCAGGAATACTTACGTCGTTATGGTTTAGTGAAGTGGTCAATGT<br>HRC2-VB1-mRgs5-485: AGGTTCTTCATGGTGATGTCTTATAGGTCGAGTAGTATAGCCAGGTT<br>HLC2-VB1-mRgs5-634: AGGTCAGGAATACTTACGTCGTTATGGTGACCAGATGACTACTTGAT<br>HRC2-VB1-mRgs5-634: CAGGGCAACTTTTGGAAGCCTTATAGGTCGAGTAGTATAGCCAGGTT<br>HLC2-VB1-mRgs5-708: AGGTCAGGAATACTTACGTCGTTATGGTGTGCAGGTGCCTGGGAGGA<br>HRC2-VB1-mRgs5-708: GCAAAGCTGCTATGGAGAACTTATAGGTCGAGTAGTATAGCCAGGTT<br>HLC2-VB1-mRgs5-789: AGGTCAGGAATACTTACGTCGTTATGGTAACATGAGCGAAAGAGTT<br>HRC2-VB1-mRgs5-789: ACAGATCCACATAACCAAACTTATAGGTCGAGTAGTATAGCCAGGTT<br>HLC2-VB1-mRgs5-885: AGGTCAGGAATACTTACGTCGTTATGGTGACAATGACTTGGTAAATC<br>HRC2-VB1-mRgs5-885: TTTATTACAAAGCAGTCAGAATTATAGGTCGAGTAGTATAGCCAGGTT
Vegfa
HL-VB1-mVegfa-98: AGGTCAGGAATACTTACGTCGTTATGGTTGTCGCTGCGTCTCGCGA<br>HR-VB1-mVegfa-98: AGTCCGCTGAATAGTCTGCCTTATAGGTCGAGTAGTATAGCCAGGTT<br>HL-VB1-mVegfa-298: AGGTCAGGAATACTTACGTCGTTATGGTGATTTCCACAATCCGAAGT<br>HR-VB1-mVegfa-298: TTGATACCTCTTTCGTCTGCTTATAGGTCGAGTAGTATAGCCAGGTT<br>HL-VB1-mVegfa-352: AGGTCAGGAATACTTACGTCGTTATGGTCTCTCTCTCTTCCTTGACT<br>HR-VB1-mVegfa-352: GCGCTCTCTCTGACCGGTCTTATAGGTCGAGTAGTATAGCCAGGTT<br>HL-VB1-mVegfa-437: AGGTCAGGAATACTTACGTCGTTATGGTGGCGGTCACCCCCAAAAGC<br>HR-VB1-mVegfa-437: AGGGCTCACGCCGCGCTCTTATAGGTCGAGTAGTATAGCCAGGTT<br>HL-VB1-mVegfa-481: AGGTCAGGAATACTTACGTCGTTATGGTGGTCCGATGCAAGATCCCA<br>HR-VB1-mVegfa-481: TGTCTGTCCGTCAGCGCGACTTATAGGTCGAGTAGTATAGCCAGGTT<br>HL-VB1-mVegfa-587: AGGTCAGGAATACTTACGTCGTTATGGTTCGGTTCCTCGCGGCTCG<br>HR-VB1-mVegfa-587: CCCCGCCTCCGGGCGCGGGCTTATAGGTCGAGTAGTATAGCCAGGTT<br>HL-VB1-mVegfa-679: AGGTCAGGAATACTTACGTCGTTATGGTACGGAGCGAGAAGAGCCCA<br>HR-VB1-mVegfa-679: CGGCGCAGACCACGGCTACTTATAGGTCGAGTAGTATAGCCAGGTT
Th

HLC2-VB3-TH-131: AGGTCAGGAATACCAGGTTGTAATGGTTGGGGACGTGACAGCCTCG<br>HRC2-VB3-TH-131: CTGCCGCCGTCCAATGAACCTTAGTATGATGACAATATAGCCAGGTT<br>HLC2-VB3-TH-152: AGGTCAGGAATACCAGGTTGTAATGGTCTGCCGCCGTCCAATGAAC<br>HRC2-VB3-TH-152: GCGGGCATCCTCGATGAGACTTAGTATGATGACAATATAGCCAGGTT<br>HLC2-VB3-TH-626: AGGTCAGGAATACCAGGTTGTAATGGTGGTCAGAGAAGCCCGGATG<br>HRC2-VB3-TH-626: CGGCGCTGGCGATACGCCTTAGTATGATGACAATATAGCCAGGTT<br>HLC2-VB3-TH-804: AGGTCAGGAATACCAGGTTGTAATGGTGGAAAGCCTCCAGGTGTTC<br>HRC2-VB3-TH-804: ACAGTACCGTTCCAGAAGCTTAGTATGATGACAATATAGCCAGGTT<br>HLC2-VB3-TH-1011: AGGTCAGGAATACCAGGTTGTAATGGTCGGGTGAGTGCATAGGTGA<br>HRC2-VB3-TH-1011: CTCGTGGCAGCAGTCTGGCTTAGTATGATGACAATATAGCCAGGTT<br>HLC2-VB3-mTH-1448: AGGTCAGGAATACCAGGTTGTAATGGTGTCCAGTACATCAATGGCC<br>HRC2-VB3-mTH-1448: CGCCGGATGGTGTGAGGACTTAGTATGATGACAATATAGCCAGGTT<br>HLC2-VB3-mTH-1707: AGGTCAGGAATACCAGGTTGTAATGGTGGAGCGCATGCAGTAGTAA<br>HRC2-VB3-mTH-1707: GGAGGAATGCAGGACCATCCTTAGTATGATGACAATATAGCCAGGTT
Cyp11a1
HL-VB2-CYP11a1-148: AGGTCAGGAATACTTAGCTATTGATGGTCAGGAAAGGTTGGCAGCCT<br>HR-VB2-CYP11a1-148: GGACCCTGCCACGTAGGGCTTCTGTGTAGACGACTATAGCCAGGTT<br>HL-VB2-CYP11a1-209: AGGTCAGGAATACTTAGCTATTGATGGTGCTAGTAGAGGTACCAGC<br>HR-VB2-CYP11a1-209: CTCATTGAAGGACCTAGGACTTCTGTGTAGACGACTATAGCCAGGTT<br>HL-VB2-CYP11a1-265: AGGTCAGGAATACTTAGCTATTGATGGACAGGTTTAGCCAACCATTG<br>HR-VB2-CYP11a1-265: CCACCCCTCCAGAAGTGGTTTCTGTGTAGACGACTATAGCCAGGTT<br>HL-VB2-CYP11a1-320: AGGTCAGGAATACTTAGCTATTGATGGTCTGCATCTGATGGTAATG<br>HR-VB2-CYP11a1-320: GGGGCCATACTTTTGGAAACTTCTGTGTAGACGACTATAGCCAGGTT<br>HL-VB2-CYP11a1-392: AGGTCAGGAATACTTAGCTATTGATGGTTGGGGTCCACGATGTAAAC<br>HR-VB2-CYP11a1-392: AGAAGAGTATCGACGCATCCTTCTGTGTAGACGACTATAGCCAGGTT

Reference

FIELDING, J. W., HODSON, E. J., CHENG, X., FERGUSON, D. J. P., ECKARDT, L., ADAM, J., LIP, P., MATON-HOWARTH, M., RATNAYAKA, I., PUGH, C. W., *et al.* 2018. PHD2 inactivation in Type I cells drives HIF-2alpha-dependent multilineage hyperplasia and the formation of paraganglioma-like carotid bodies. *J Physiol*.
