# Supplementary material for: Developmental role of PHD2 in the pathogenesis of pseudohypoxic pheochromocytoma
Source: Endocr Relat Cancer. 2021 Sep 20;28(12):757–72. doi: 10.1530/ERC-21-0211 (PMC8558849; doi:10.1530/ERC-21-0211)
Supplement: Supplementary Table 1. Genes commonly dysregulated in pseudohypoxic PPGLs. Genes from several classes (HIF target genes, G protein signalling pathway components, atypical mitochondrial subunits and genes with oncogenic potential) which are commonly dysregulated in pseudohypoxic PPGLs were identified [file supplementary_table_1.pdf]

***Supplementary Table 1*. Genes commonly dysregulated in pseudohypoxic PPGLs.** Genes from several classes (HIF target genes, G protein signalling pathway components, atypical mitochondrial subunits and genes with oncogenic potential) which are commonly dysregulated in pseudohypoxic PPGLs were identified from human studies, as detailed below.

**Gene**	**Differential regulation**	**Mutation and type of PPGL**	**Reference**
VEGFA	Upregulated	*VHL*- and *SDHx*-PCCs	Dahia *et al*., 2005
		SDHx- and *VHL*-PPGLs	Burnichon *et al.*, 2011
		VHL- and *SDHx*-PPGLs and *HIF-2α*-PCCs	Favier *et al*., 2012
		HIF-2α-PGLs	Lorenzo *et al*., 2013
		HIF-2α-PPGLs	Toledo *et al*., 2013
		HIF-2α-PCCs	Welander *et al*., 2014
		PHD1- and *PHD2*-PCCs	Yang *et al*., 2015
SLC2A1	Upregulated	*VHL*- and *SDHx*-PPGLs	Favier *et al*., 2009
		VHL-PPGLs	Burnichon *et al*., 2011
		HIF-2α-PGL	Lorenzo *et al*., 2013
		PHD2-PCCs	Yang *et al*., 2015
CCND1	Not dysregulated	Pseudohypoxic (*VHL*, *SDH*x) PPGLs	Toledo *et al*., 2013
		HIF-2α-PCCs	Welander *et al*., 2014
	Upregulated	*HIF-2α*-PPGLs	Toledo *et al*., 2013
LDHA	Upregulated	*VHL*- and *SDHx*-PPGLs	Dahia *et al*., 2005
		VHL- and *SDHx*-PPGLs	Favier *et al*., 2009
		SDHx- and *VHL*-PPGLs	Burnichon *et al.*, 2011
		PHD1- and *PHD2*-PPGLs	Yang *et al*., 2015
HIF-2α	Upregulated	*SDHx*- and *VHL*-PPGLs	Burnichon *et al.*, 2011
		VHL- and *SDHx*-PPGLs	Favier *et al*., 2009
		VHL- and *SDHx*-PPGLs and *HIF-2α*-PCCs	Favier *et al*., 2012
		HIF-2α-PCCs	Welander *et al*., 2014
NDUFA4L2	Upregulated	*VHL*- and *SDHx*-PPGLs	Favier *et al*., 2009
		SDHx- and *VHL*-PPGLs	Burnichon *et al.*, 2011
		Pseudohypoxic (*SDHx/VHL/HIF-2α/ PHD2*) PPGLs	Fishbein *et al*., 2017
COX4I2	Upregulated	*VHL*- and *SDHx*-PPGLs	Favier *et al*., 2009
		SDHx- and *VHL*-PPGLs	Burnichon *et al.*, 2011
		VHL- and *SDHx*-PPGLs and *HIF-2α*-PCCs	Favier *et al*., 2012
		HIF-2α-PCCs	Welander *et al*., 2014
		Pseudohypoxic (*SDHx/VHL/HIF-2α/ PHD2*) PPGLs	Fishbein *et al*., 2017
RGS4	Upregulated	*SDHx*- and *VHL*-PPGLs	Burnichon *et al.*, 2011
RGS5	Upregulated	PPGLs	Waldmann *et al.*, 2010
ADORA2A	Upregulated	*SDHx*- and *VHL*-PPGLs	Burnichon *et al.*, 2011
PNMT	Downregulated	*VHL*- and *SDHx*-PPGLs	Dahia *et al*., 2005
		HIF-2α-PCCs	Welander *et al*., 2014
		Pseudohypoxic (*SDHx/VHL/HIF-2α/*	Fishbein *et al*., 2017

		PHD2) PPGLs	
STC1	Upregulated	*VHL*- and *SDHx*-PPGLs	Dahia *et al*., 2005
		SDHx- and *VHL*-PPGLs	Burnichon *et al.*, 2011
IGF2	Upregulated	PPGLs	Waldmann *et al.*, 2010
		HIF-2α-PPGLs	Toledo *et al*., 2013
RET	Downregulated	*VHL*- and *SDHx*-PPGLs	Dahia *et al*., 2005
		Pseudohypoxic (*SDHx/VHL/HIF-2α/ PHD2*) PPGLs	Fishbein *et al*., 2017

References

BURNICHON, N., VESCOVO, L., AMAR, L., LIBE, R., DE REYNIES, A., VENISSE, A., JOUANNO, E., LAURENDEAU, I., PARFAIT, B., BERTHERAT, J.*, et al.* 2011. Integrative genomic analysis reveals somatic mutations in pheochromocytoma and paraganglioma. *Hum Mol Genet,* 20**,** 3974-85.

DAHIA, P. L., ROSS, K. N., WRIGHT, M. E., HAYASHIDA, C. Y., SANTAGATA, S., BARONTINI, M., KUNG, A. L., SANSO, G., POWERS, J. F., TISCHLER, A. S.*, et al.* 2005. A HIF1alpha regulatory loop links hypoxia and mitochondrial signals in pheochromocytomas. *PLoS Genet,* 1**,** 72-80.

FAVIER, J., BRIÈRE, J. J., BURNICHON, N., RIVIÈRE, J., VESCOVO, L., BENIT, P., GISCOS-DOURIEZ, I., DE REYNIÈS, A., BERTHERAT, J., BADOUAL, C.*, et al.* 2009. The Warburg effect is genetically determined in inherited pheochromocytomas. *PLoS One,* 4**,** e7094.

FAVIER, J., BUFFET, A. & GIMENEZ-ROQUEPLO, A. P. 2012. HIF2A mutations in paraganglioma with polycythemia. *N Engl J Med,* 367**,** 2161; author reply 2161-2.

FISHBEIN, L., LESHCHINER, I., WALTER, V., DANILOVA, L., ROBERTSON, A. G., JOHNSON, A. R., LICHTENBERG, T. M., MURRAY, B. A., GHAYEE, H. K., ELSE, T.*, et al.* 2017. Comprehensive Molecular Characterization of Pheochromocytoma and Paraganglioma. *Cancer Cell,* 31**,** 181-193.

LORENZO, F. R., YANG, C., NG TANG FUI, M., VANKAYALAPATI, H., ZHUANG, Z., HUYNH, T., GROSSMANN, M., PACAK, K. & PRCHAL, J. T. 2013. A novel EPAS1/HIF2A germline mutation in a congenital polycythemia with paraganglioma. *J Mol Med (Berl),* 91**,** 507-12.

TOLEDO, R. A., QIN, Y., SRIKANTAN, S., MORALES, N. P., LI, Q., DENG, Y., KIM, S. W., PEREIRA, M. A., TOLEDO, S. P., SU, X.*, et al.* 2013. In vivo and in vitro oncogenic effects of HIF2A mutations in pheochromocytomas and paragangliomas. *Endocr Relat Cancer,* 20**,** 349-59.

WALDMANN, J., FENDRICH, V., HOLLER, J., BUCHHOLZ, M., HEINMÖLLER, E., LANGER, P., RAMASWAMY, A., SAMANS, B., WALZ, M. K., ROTHMUND, M.*, et al.* 2010. Microarray analysis reveals differential expression of benign and malignant pheochromocytoma. *Endocr Relat Cancer,* 17**,** 743-56.

WELANDER, J., ANDREASSON, A., BRAUCKHOFF, M., BACKDAHL, M., LARSSON, C., GIMM, O. & SODERKVIST, P. 2014. Frequent EPAS1/HIF2alpha exons 9 and 12 mutations in non-familial pheochromocytoma. *Endocr Relat Cancer,* 21**,** 495-504.

YANG, C., ZHUANG, Z., FLIEDNER, S. M., SHANKAVARAM, U., SUN, M. G., BULLOVA, P., ZHU, R., ELKAHLOUN, A. G., KOURLAS, P. J., MERINO, M.*, et al.* 2015. Germ-line PHD1 and PHD2 mutations detected in patients with pheochromocytoma/paraganglioma-polycythemia. *J Mol Med (Berl),* 93**,** 93-104.
