# Supplementary material for: Developmental role of PHD2 in the pathogenesis of pseudohypoxic pheochromocytoma
Source: Endocr Relat Cancer. 2021 Sep 20;28(12):757–72. doi: 10.1530/ERC-21-0211 (PMC8558849; doi:10.1530/ERC-21-0211)
Supplement: Supplementary Table 2. Volumes and proliferation of AMs with constitutive and adult-onset Phd2 inactivation. Quantification of AM volumes and Ki67+ cells per mm2 of AMs in Phd2f/f;THCre, Phd2f/f;THCreER, Phd2f/f;RosaCreER mice and their littermate controls (Phd2f/f). Data were analysed by Student’s  [file supplementary_table_2.pdf]

Supplementary Table 2. **Volumes and proliferation of AMs with constitutive and adult-onset *Phd2* inactivation.** Quantification of AM volumes and $Ki67^+$ cells per mm^2 of AMs in *$Phd2^{f/f}$;THCre*, *$Phd2^{f/f}$;THCreER, $Phd2^{f/f}$;RosaCreER* mice and their littermate controls (*$Phd2^{f/f}$*). Data were analysed by Student's two tailed *t*-tests and are shown as mean±SEM; n=3 for all comparisons except volume in *$Phd2^{f/f}$;THCre* versus control, where n=5. No significant differences in AM volume or proliferation were noted in pairwise comparisons between *Phd2* knock-out versus control mice in any of the groups, although there were differences in values (including controls) between the paired comparisons which may be due to small differences in methods of tissue preparation.

**Mouse model**	**AM volume (mm^3)**			**$Ki67^+$ cells per mm^2 AM**		
	**Control**	**Knock-out**	***P* value**	**Control**	**Knock-out**	***P* value**
$Phd2^{f/f}$;THCre	0.08±0.01	0.08±0.02	0.731	67±30	39±21	0.495
$Phd2^{f/f}$;THCreER	0.12±0.01	0.14±0.02	0.291	17±5	17±5	0.989
$Phd2^{f/f}$;RosaCreER	0.15±0.00	0.16±0.01	0.432	14±7	24±10	0.429
