# Supplementary figures and images for: Developmental role of PHD2 in the pathogenesis of pseudohypoxic pheochromocytoma
Source: Endocr Relat Cancer. 2021 Sep 20;28(12):757–72. doi: 10.1530/ERC-21-0211 (PMC8558849; doi:10.1530/ERC-21-0211)

*Phd2<sup>f/f</sup>*

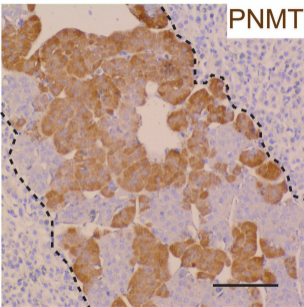

*Phd2<sup>f/f</sup>;THCre*

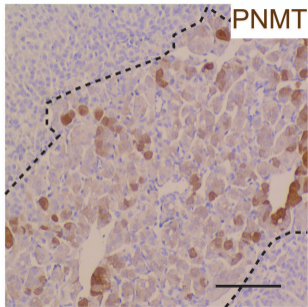

*Supplementary Figure 1*

Supplement: Supplementary Figure 1. Effect of Phd2 inactivation on PNMT protein in AMs. PNMT immunohistochemistry (brown) in the AMs of Phd2f/f and Phd2f/f;THCre mice. AMs are outlined by a black dashed line in this and other figures. Images show loss of PNMT protein after Phd2 inactivation. Harris hematoxylin  [file supplementary_figure_1.pdf]

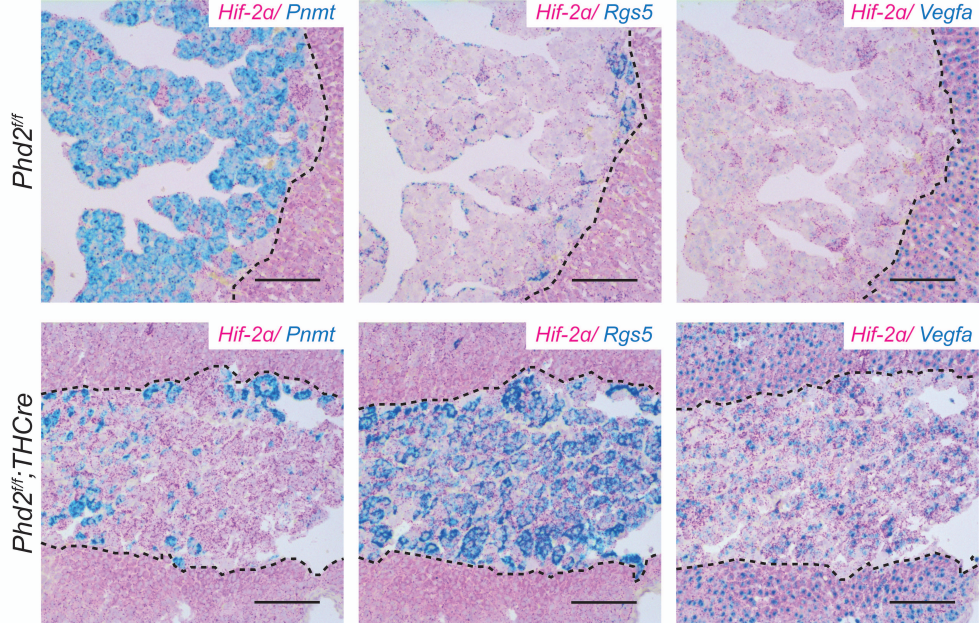

Supplementary Figure 2

Supplement: Supplementary Figure 2. Effects of TH-restricted Phd2 inactivation on spatial expression of genes in the AM. Representative images of in situ hybridisation for Hif-2α (pink) together with Pnmt, Rgs5 or Vegfa (blue) mRNA in AMs of Phd2f/f and Phd2f/f;THCre mice. Hif-2α expression in cells inversely c [file supplementary_figure_2.pdf]

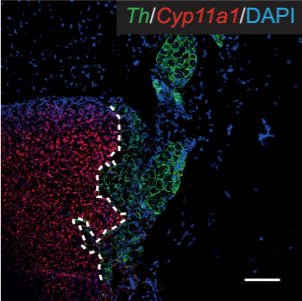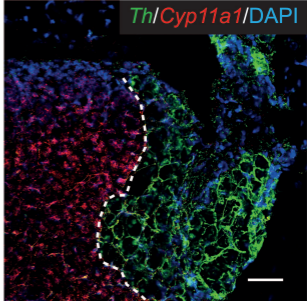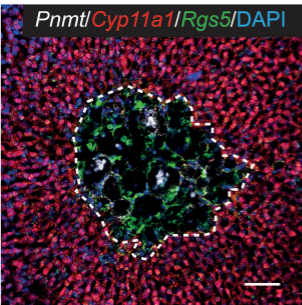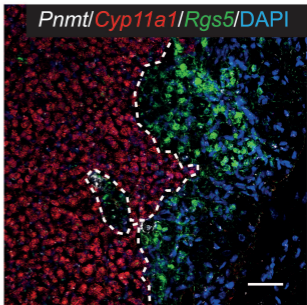

Supplementary Figure 3

Supplement: Supplementary Figure 3. Adrenal cortical marker expression in adrenal glands of Phd2f/f;THCre mice. In situ detection of the adrenal cortical marker Cyp11a1 (red), Th (green, top panels), Pnmt (white, bottom panels) and Rgs5 (green, bottom panels) in the adrenal glands of Phd2f/f;THCre mice with bot [file supplementary_figure_3.pdf]

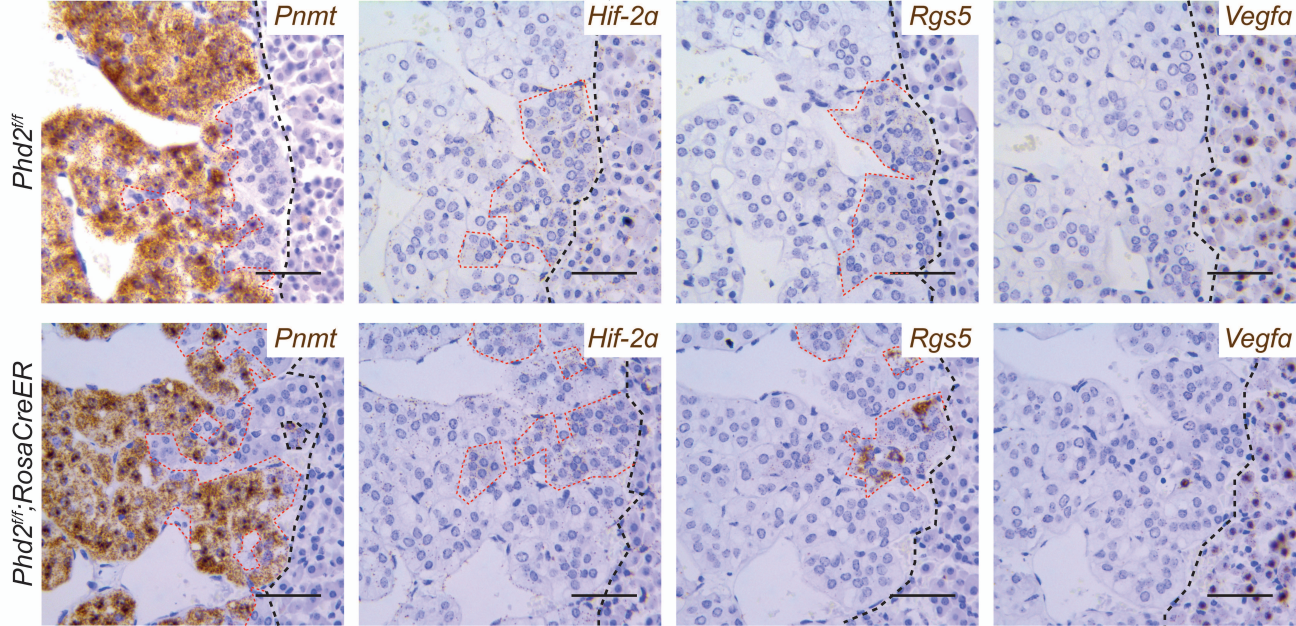

Supplementary Figure 4

Supplement: Supplementary Figure 4. Spatial distribution of gene expression in the AM with ubiquitous, adult-onset Phd2 inactivation. Representative images of in situ hybridisation for Pnmt, Hif-2α, Rgs5 and Vegfa mRNA in the AMs of Phd2f/f;RosaCreER mice and their littermate controls. Pnmt-/Hif-2α+/Rgs5+ area  [file supplementary_figure_4.pdf]

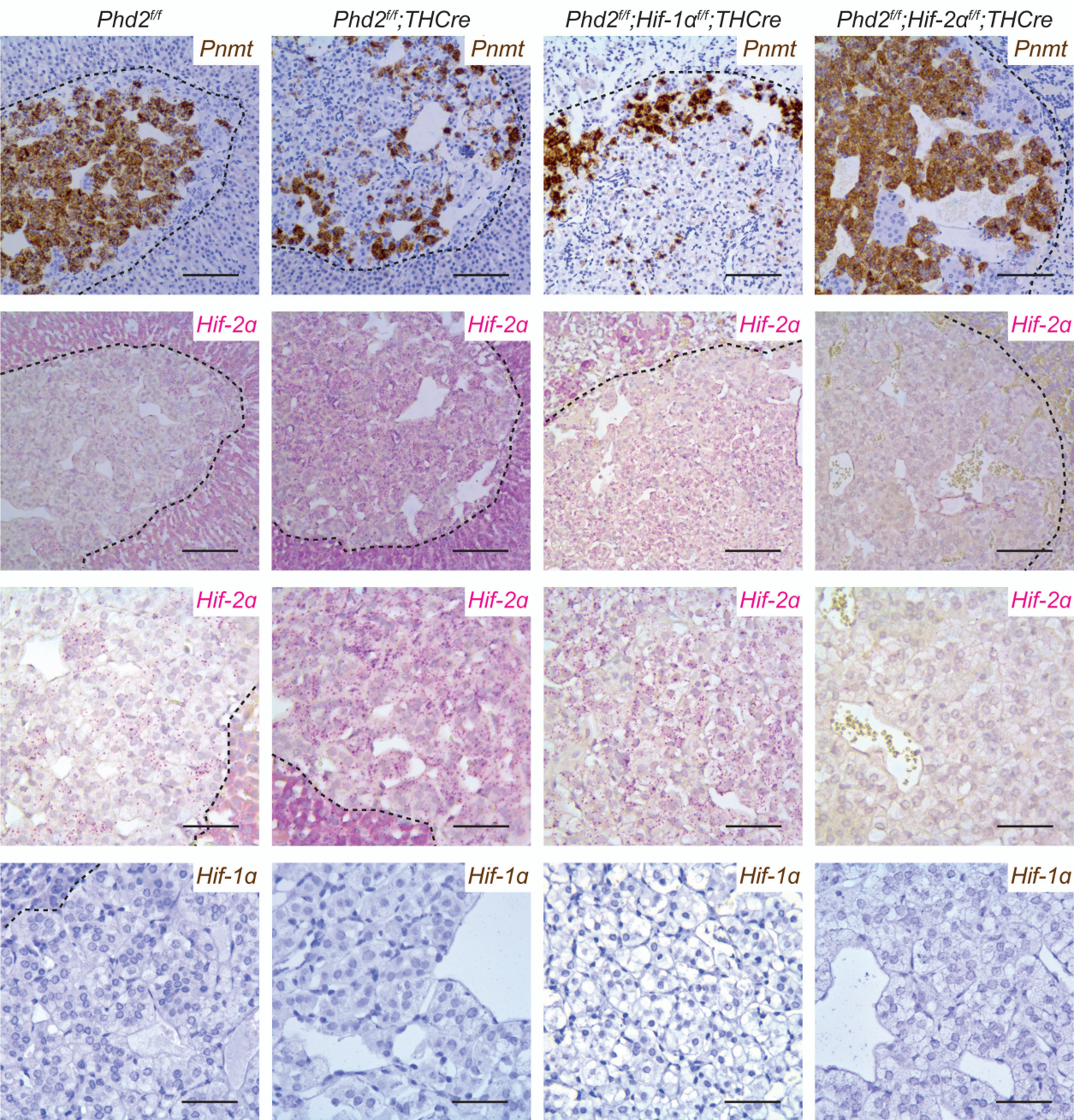

Supplementary Figure 5

Supplement: Supplementary Figure 5. Hif-1/2α expression in the AMs of mice with concomitant Phd2 and Hif-1/2α inactivation. Representative images of in situ hybridisation for Pnmt, Hif-2α and Hif-1α in the AMs of Phd2f/f, Phd2f/f;THCre, Phd2f/f;Hif-1αf/f;THCre and Phd2f/f;Hif-2αf/f;THCre mice. Loss of Hif-2α mR [file supplementary_figure_5.pdf]

*Phd2<sup>fl/fl</sup>*

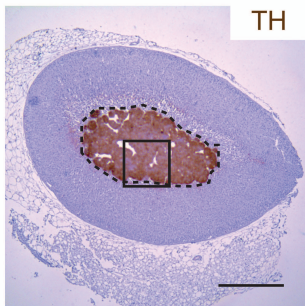

*Phd2<sup>fl/fl</sup>;Hif-1α<sup>fl/fl</sup>;THCre*

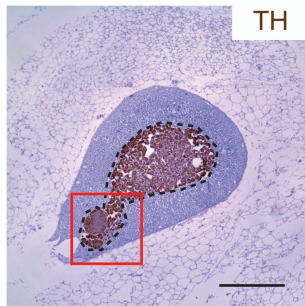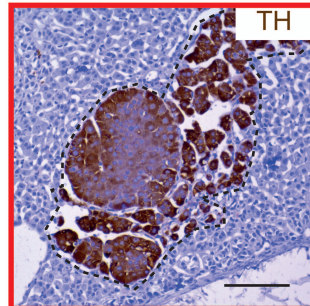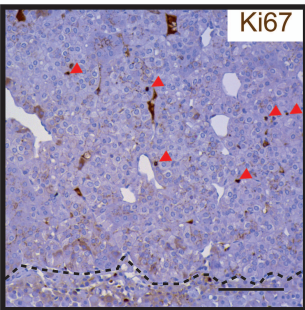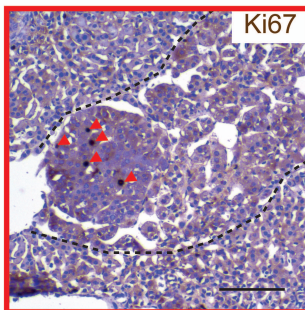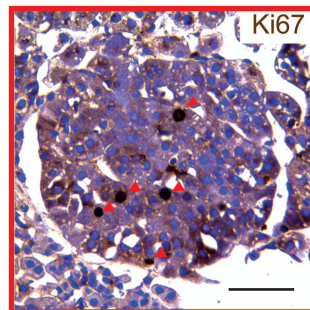

Supplementary Figure 6

Supplement: Supplementary Figure 6. TH+ nodule in the AM of an aged mouse with concomitant inactivation of Phd2 and Hif-1α. TH and Ki67 immunohistochemistry in the AMs of ~18 month-old Phd2f/f;Hif-1αf/f;THCre and littermate control (Phd2f/f) mice. Higher magnification images denoted with: a black box in the con [file supplementary_figure_6.pdf]
